# Supplementary material for: Lead halide perovskites for photocatalytic organic synthesis
Source: Nat Commun. 2019 Jun 28;10:2843. doi: 10.1038/s41467-019-10634-x (PMC6599021; doi:10.1038/s41467-019-10634-x)
Supplement: Supplementary file 1 — Supplementary Information [file 41467_2019_10634_MOESM1_ESM.pdf]

Supplementary Information

Lead-Halide Perovskites for Photocatalytic Organic Synthesis

Zhu *et al*.

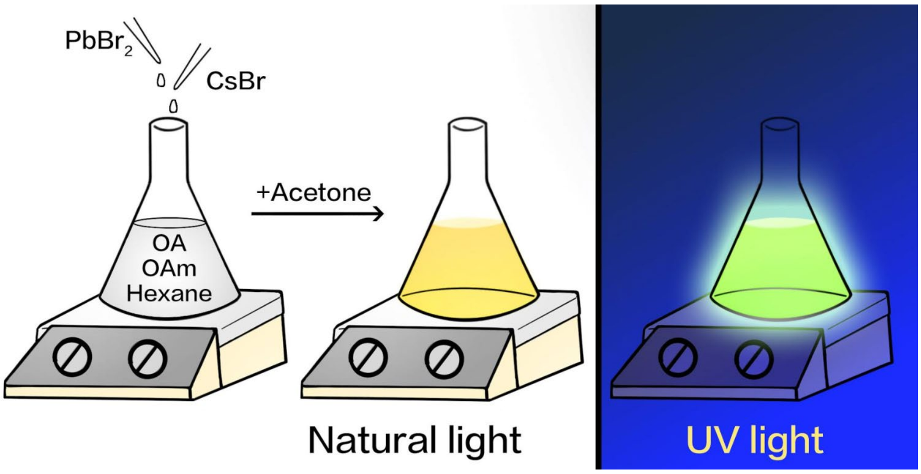

Supplementary Figure 1. The demonstration of $CsPbBr_3$ nanocrystals **P1** synthesis.

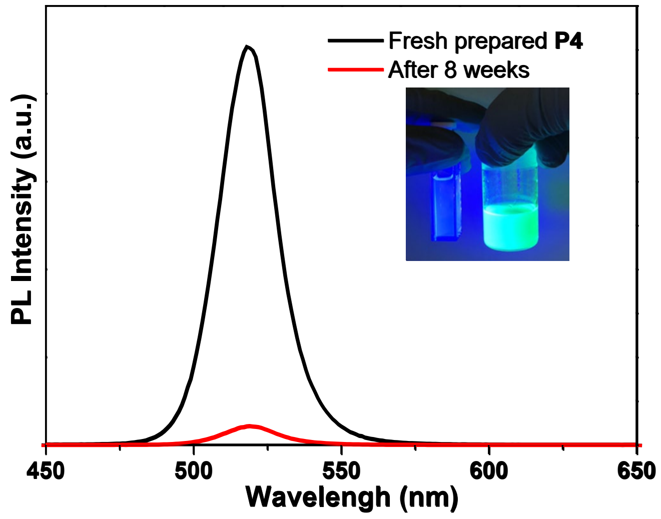

Supplementary Figure 2. The PL spectra for as-prepared $CsPbBr_3$ **P4** and after 8 weeks in Hexane. Inset: the photograph of the sample after 8 weeks (left) and as-prepared (right) in Hexane under the irradiation of 365 nm UV light (right). Source data are provided as a Source Data file.

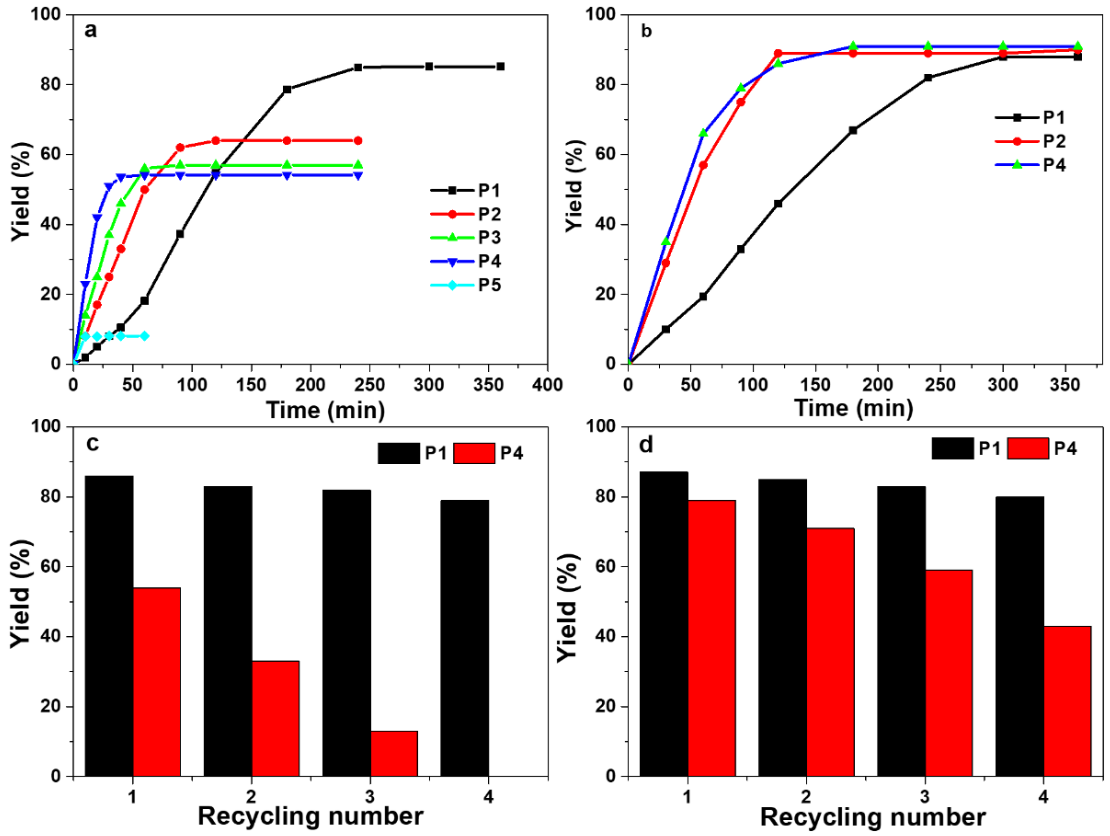

Supplementary Figure 3. The comparation yields for the synthesis of **1a** and **2a** by using different perovskites. (a) Time dependence for the perovskite **P1-P5** of reaction yields for **1a**; (b) Time dependence for the perovskite **P1**, **P2** and **P4** of reaction yields for **2a**; Reusability test for the perovskite **P1**, and **P4**, of reaction yields for **1a** (c) and **2a** (d). Yield determined by ^{1}H NMR. Source data are provided as a Source Data file.

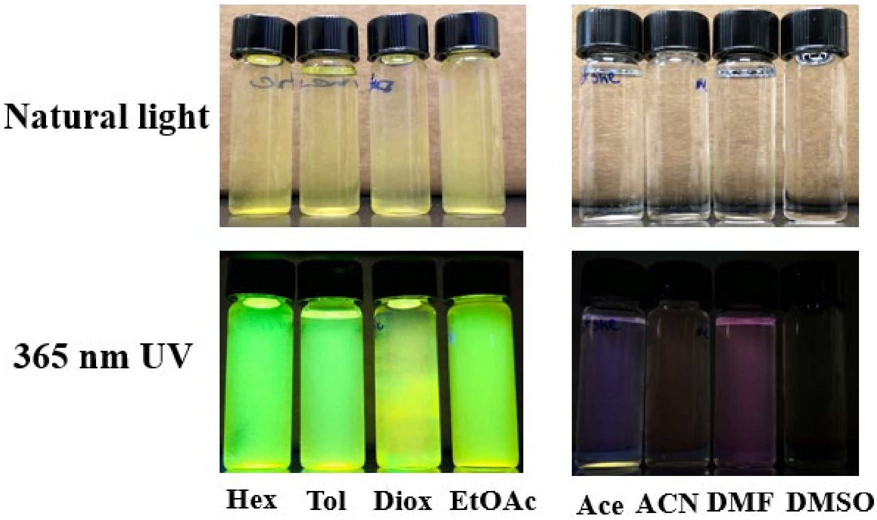

Supplementary Figure 4. The photograph for $CsPbBr_3$ **P1** disperse in different solvents. Left, less polar solvents: Hexane, Toluene, 1,4-dioxane, ethyl acetate; right, polar solvents: Acetone, acetonitrile, DMF, and DMSO at ambient light (top) and 365 nm UV light (bottom).

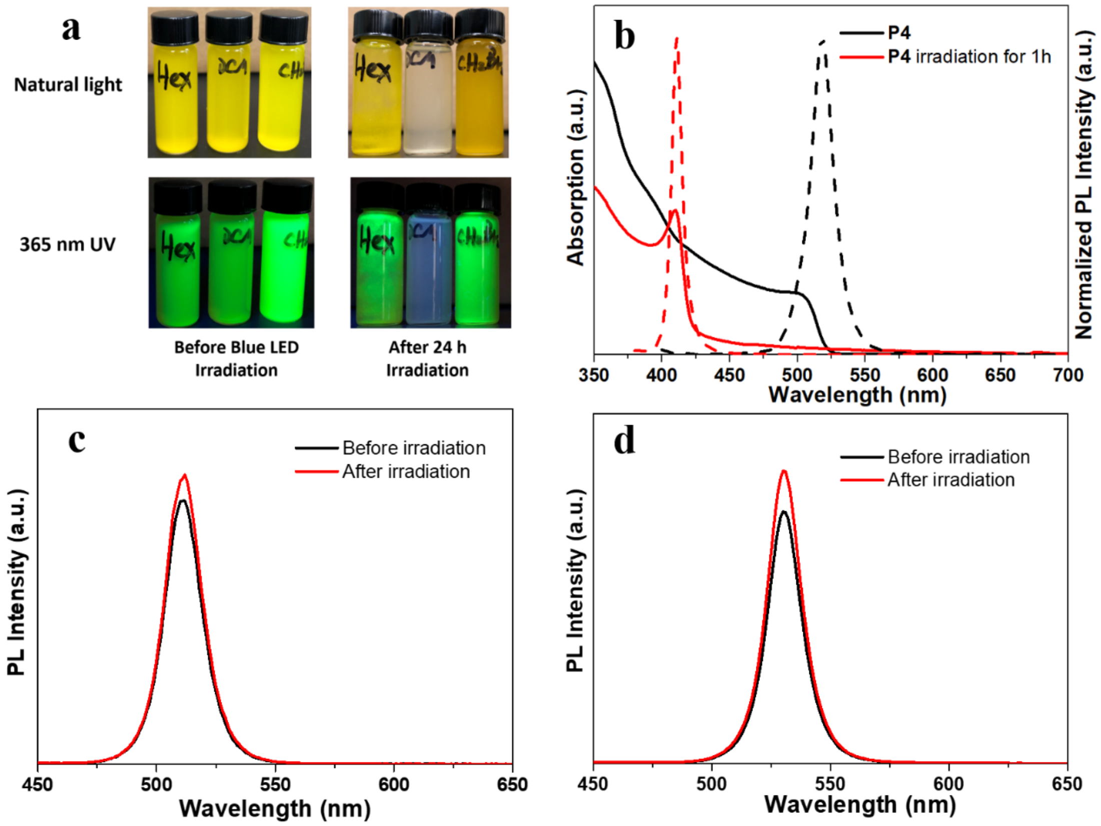

Supplementary Figure 5. The comparison of **P4** in halide or non-halide solvents. (**a**) The photograph for $CsPbBr_3$ **P4** before and after the irradiation of blue LED for 2h in Hexane, CH_2Cl_2 and CH_2Br_2 at ambient light (top) and 365 nm UV light (bottom); (**b**) The UV-vis and PL spectra for **P4** before the irradiation of LED and after irradiation in CH_2Cl_2 for 1h; (**c**) The PL spectra for **P4** before the irradiation of LED and after irradiation in Hexane for 5h; (**d**) The PL spectra for **P4** before the irradiation of LED and after irradiation in 1,4-dioxane for 5h. Source data are provided as a Source Data file.

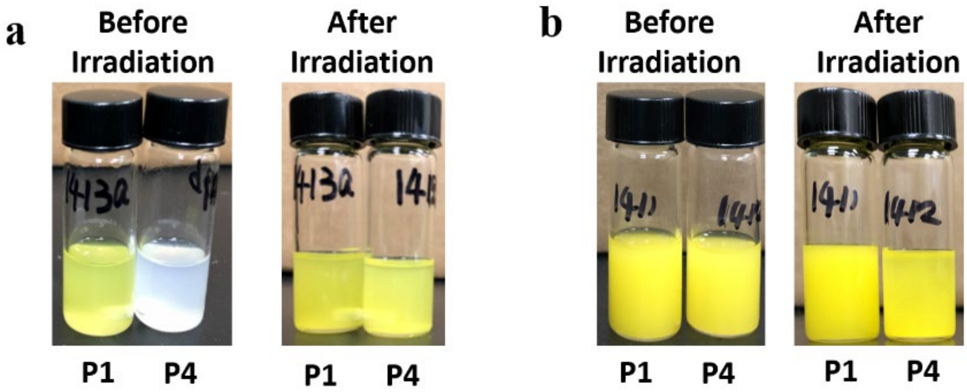

Supplementary Figure 6. Comparison of the color for different reaction mixtures. The comparison reaction mixtures of **P1** and **P4** before and after irradiation with blue LED for 6 h **(a)** in synthesis of **1a**; **(b)** in synthesis of **2a**, respectively. From supplementary Fig. 6a, pale-white is immediately formed for **P4** when co-catalyst $(ClCH_2CH_2Cl)_2NH_2Cl$ was added in reaction **1a**, while the color of reaction mixture with **P1** is still yellow. Meanwhile, for reaction **2a**, both the reaction mixtures for **P1** and **P4** are yellow.

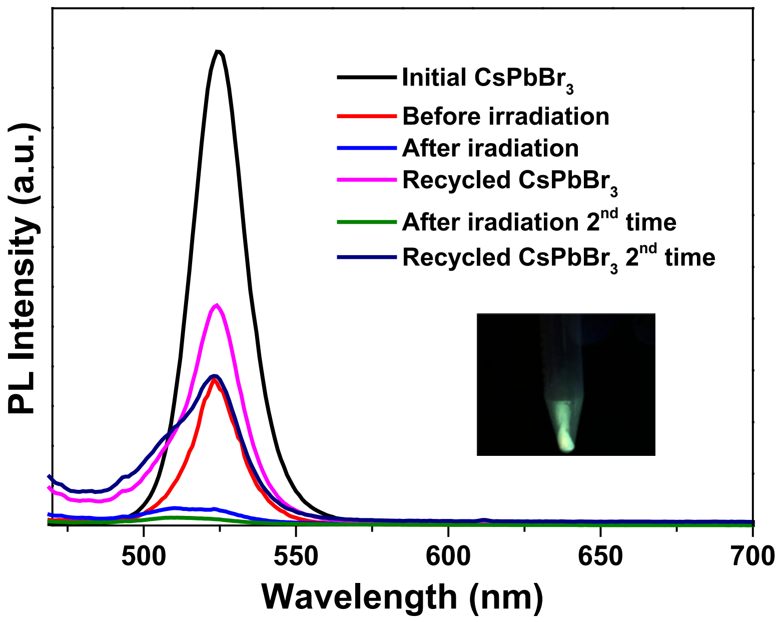

Supplementary Figure 7. The PL spectra for recycled $CsPbBr_3$ **P1**. The initial $CsPbBr_3$ NCs suspension in EtOAc (before mixing with any substrate, black line); $CsPbBr_3$ NCs suspension mixed with 1-benzylidene-2-phenylhydrazine, 2-bromoacetophenone and base before the irradiation of LED (red line); after the irradiation of LED for 12h (blue line); the recycled $CsPbBr_3$ after centrifuging the reaction mixture and re-suspension in EtOAc (purple line); the recycled $CsPbBr_3$ applied for 2nd time reaction (green line); the 2nd time recycled $CsPbBr_3$ after centrifuging the reaction mixture and re-suspension in EtOAc (violet line). Inset: photograph of the recycled $CsPbBr_3$ (mixed with base residue) after EtOAc washing under UV light. Source data are provided as a Source Data file.

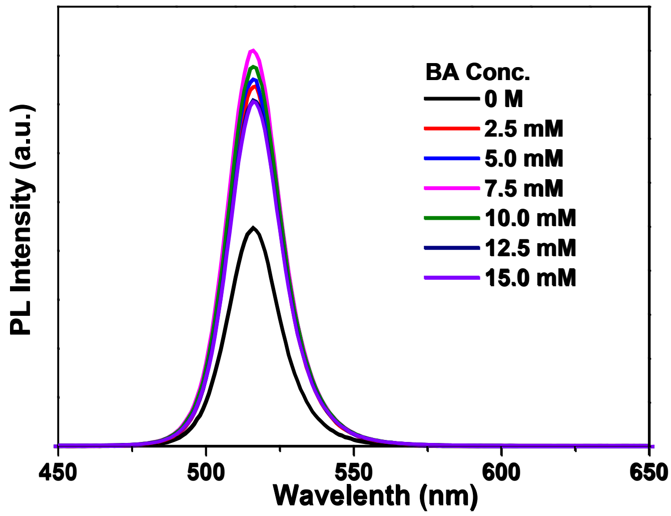

Supplementary Figure 8. PL spectra of **P1** with addition of benzoic acid. Source data are provided as a Source Data file.

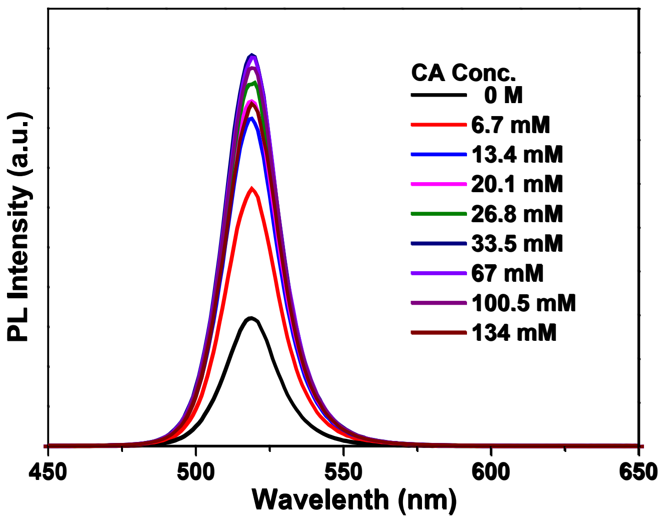

Supplementary Figure 9. PL spectra of **P1** with addition of propionic acid. Source data are provided as a Source Data file.

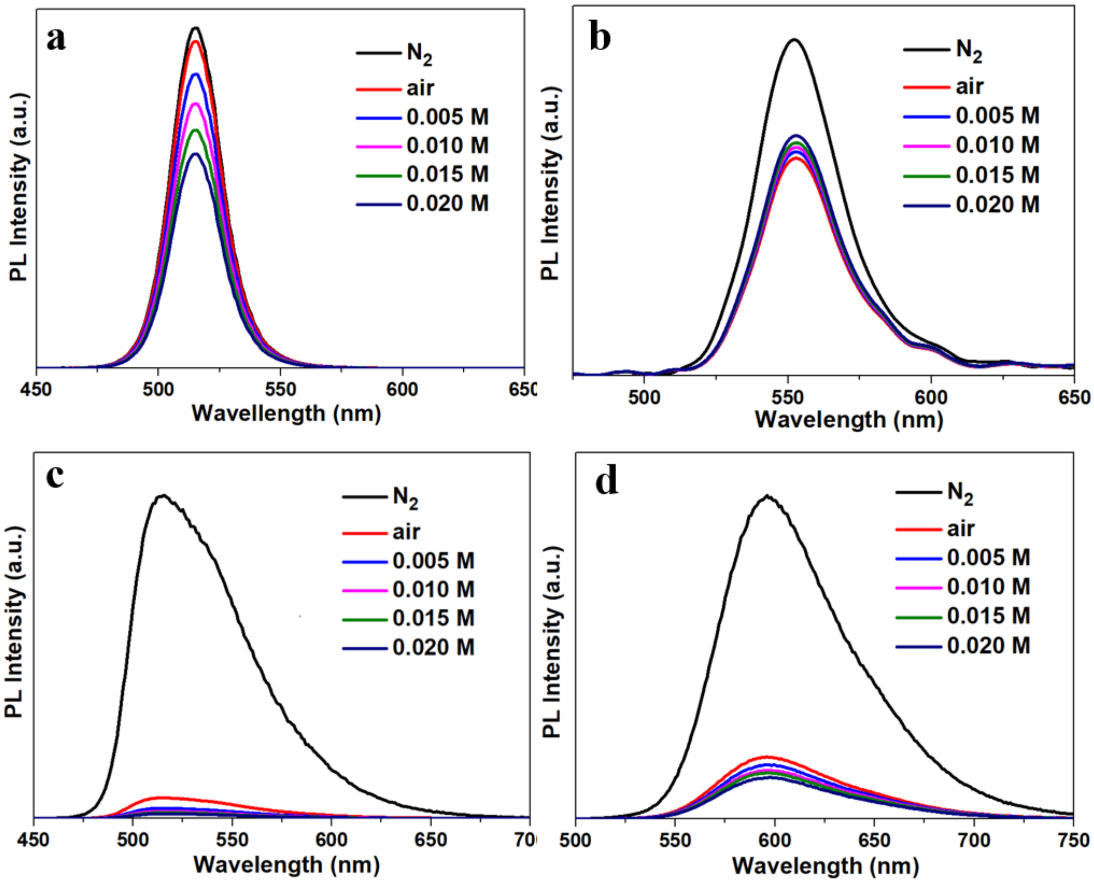

Supplementary Figure 10. Stern-Volmer quenching studies of photocatalysts with air or organic substrate $PhCOCH_2Br$. (a) $CsPbBr_3$ NCs; (b) CdSe QDs; (c) $Ir(ppy)_3$ and (d) $Ru(bpy)_3Cl_2$. Source data are provided as a Source Data file.

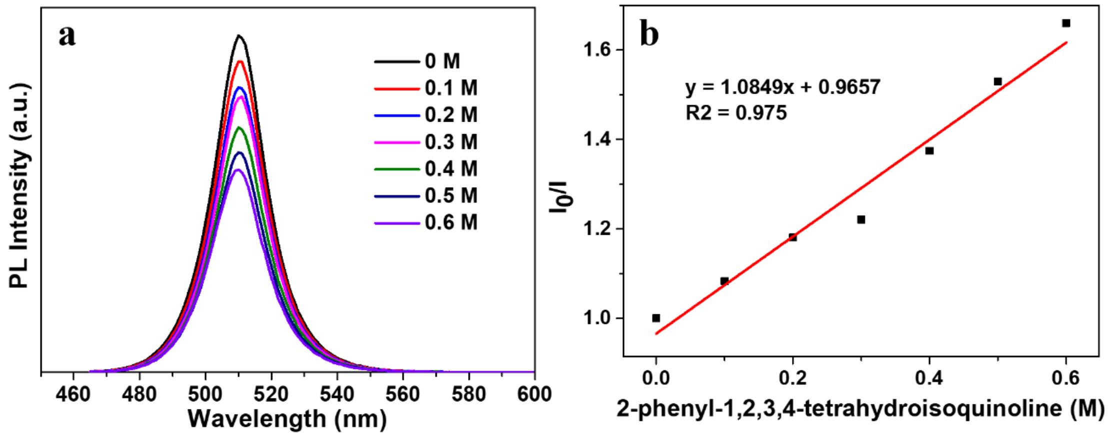

Supplementary Figure 11. $CsPbBr_3$ NCs Emission quenching by 2-phenyl-1,2,3,4-tetrahydro-isoquinoline. (a) PL spectra of $CsPbBr_3$ NCs with the addition of 2-phenyl-1,2,3,4-tetrahydro-isoquinoline. (b) Stern-Volmer quenching study of 2-phenyl-1,2,3,4-tetrahydro-isoquinoline. k_q = 1.8 $\times 10^8$ $M^{-1}s^{-1}$. Source data are provided as a Source Data file.

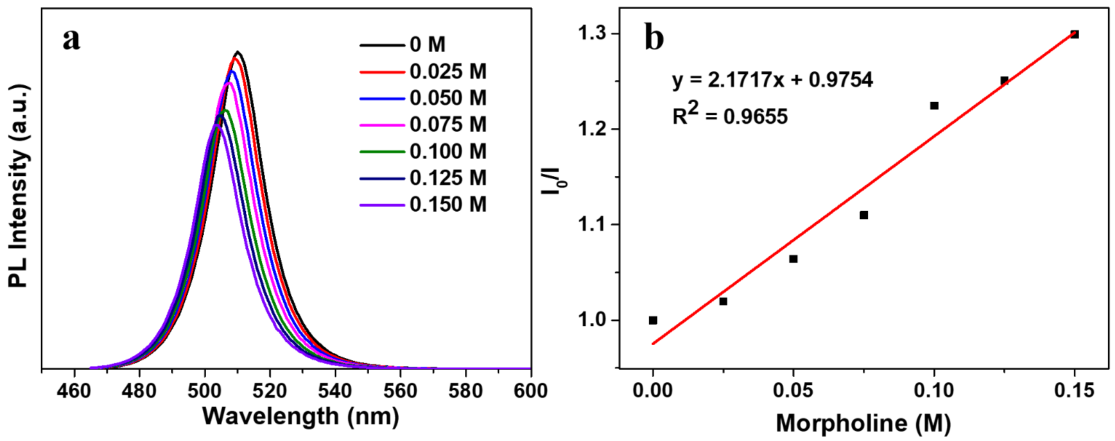

Supplementary Figure 12. $CsPbBr_3$ NCs Emission quenching by 4-phenylmorpholine. (a) PL spectra of $CsPbBr_3$ NCs with the addition of 4-phenylmorpholine. (b) Stern-Volmer quenching study of 4-phenylmorpholine. $k_q = 3.6 \times 10^8$ $M^{-1}s^{-1}$. Source data are provided as a Source Data file.

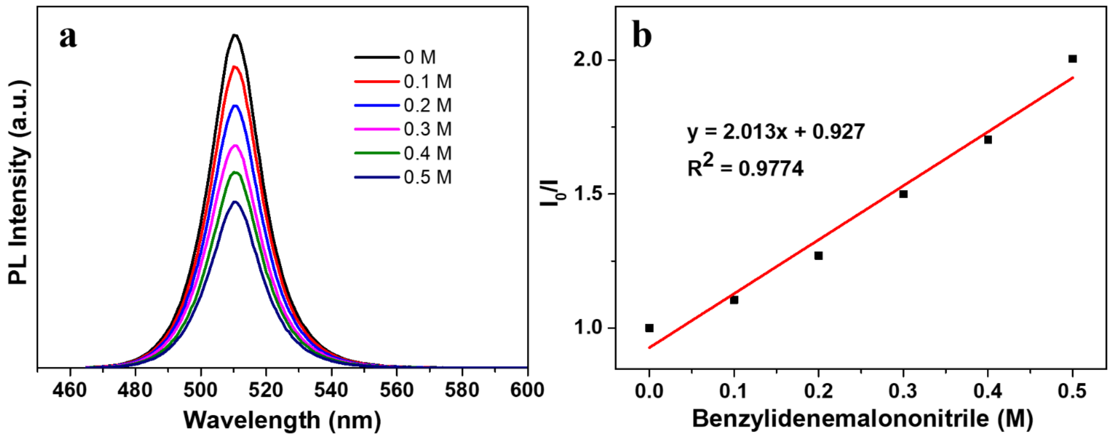

Supplementary Figure 13. $CsPbBr_3$ NCs Emission quenching by benzylidene-malononitrile. (a) PL spectra of $CsPbBr_3$ NCs with the addition of benzylidene-malononitrile. (b) Stern-Volmer quenching study of benzylidene-malononitrile. $k_q = 3.4 \times 10^8\ M^{-1}s^{-1}$. Source data are provided as a Source Data file.

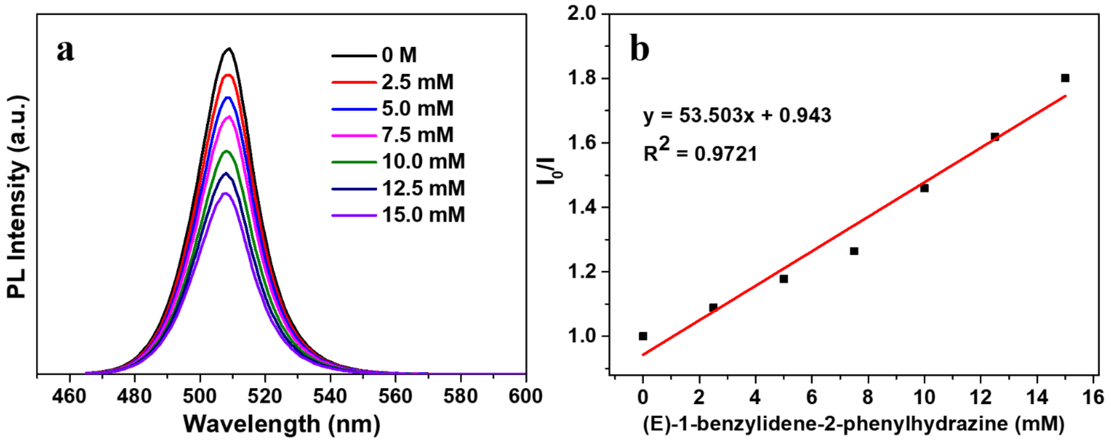

Supplementary Figure 14. $CsPbBr_3$ NCs Emission quenching by (*E*)-1-benzylidene-2-phenylhydrazine. (a) PL spectra of $CsPbBr_3$ NCs with the addition of (*E*)-1-benzylidene-2-phenylhydrazine. (b) Stern-Volmer quenching study of (*E*)-1-benzylidene-2-phenylhydrazine. $k_q = 8.8 \times 10^9$ $M^{-1}s^{-1}$. Source data are provided as a Source Data file.

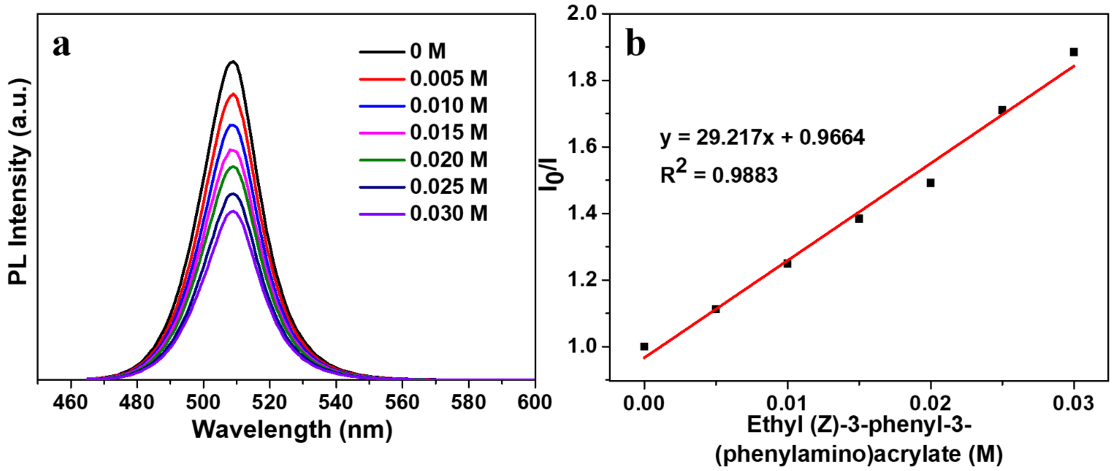

Supplementary Figure 15. $CsPbBr_3$ NCs Emission quenching by ethyl (E)-3-phenyl-3-(phenylamino)acrylate. (a) PL spectra of $CsPbBr_3$ NCs with the addition of ethyl (E)-3-phenyl-3-(phenylamino)acrylate. (b) Stern-Volmer quenching study of ethyl (E)-3-phenyl-3-(phenylamino)acrylate. $k_q = 4.9 \times 10^9$ $M^{-1}s^{-1}$. Source data are provided as a Source Data file.

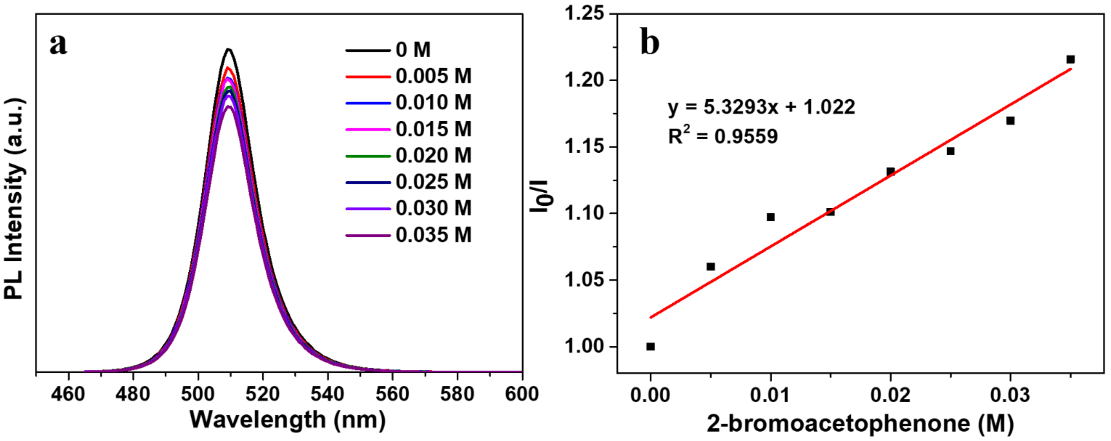

Supplementary Figure 16. $CsPbBr_3$ NCs Emission quenching by 2-bromoacetophenone in 1,4-dioxane. (a) PL spectra of $CsPbBr_3$ NCs with the addition of 2-bromoacetophenone in 1,4-dioxane. (b) Stern-Volmer quenching study of 2-bromoacetophenone in 1,4-dioxane. $k_q = 8.8 \times 10^8$ $M^{-1}s^{-1}$. Source data are provided as a Source Data file.

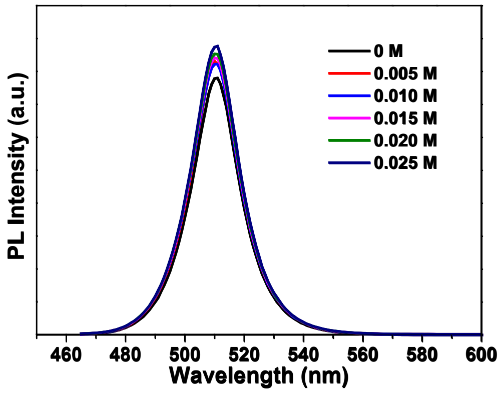

Supplementary Figure 17. $CsPbBr_3$ NCs PL changing by adding 2,4'-dichloroacetophenone. No quenching was observed, indicating the initial ET transfer is difficult corroborating with the electrochemical driving force studies. Source data are provided as a Source Data file.

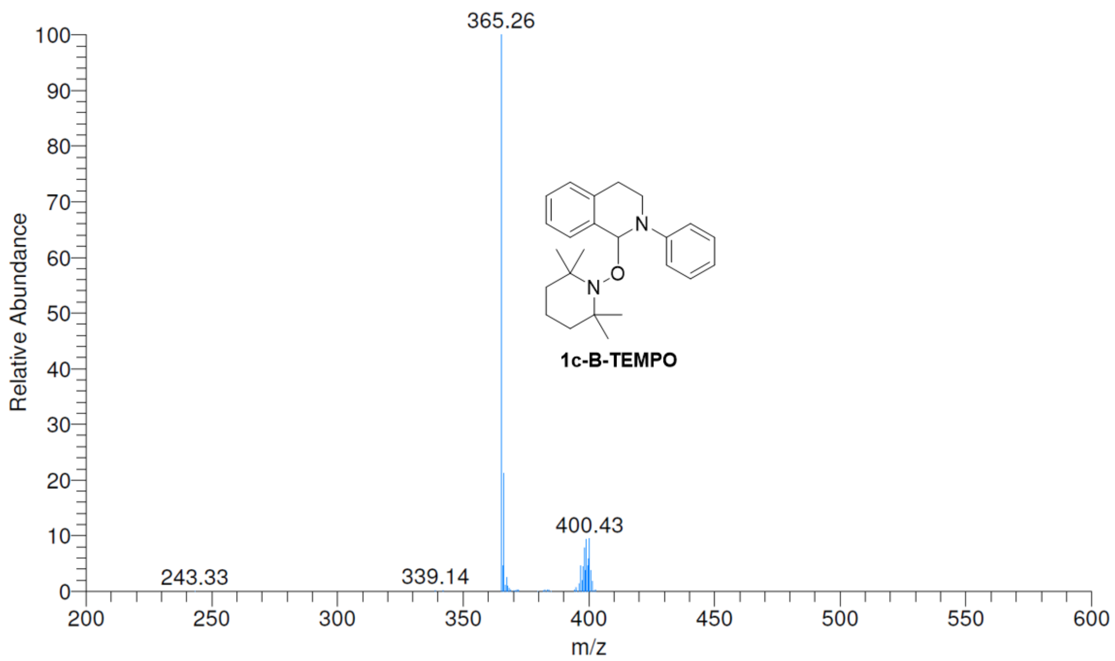

Supplementary Figure 18. LC-MS evidence for **1c-B-TEMPO**.

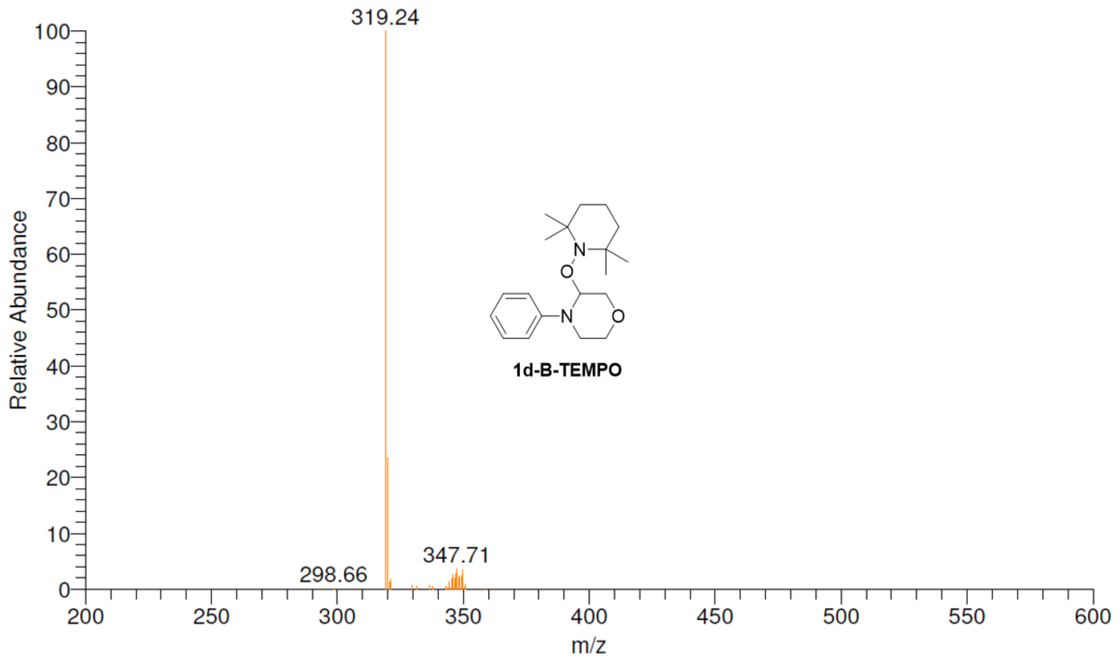

Supplementary Figure 19. LC-MS evidence for **1d-B-TEMPO**.

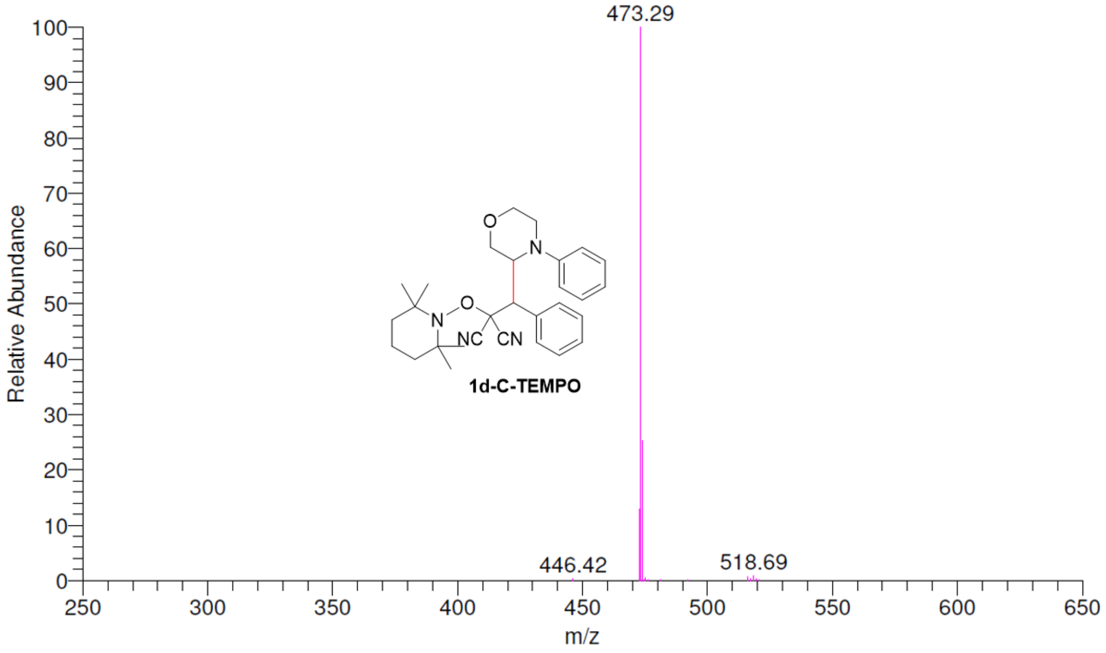

Supplementary Figure 20. LC-MS evidence for **1d-C-TEMPO**.

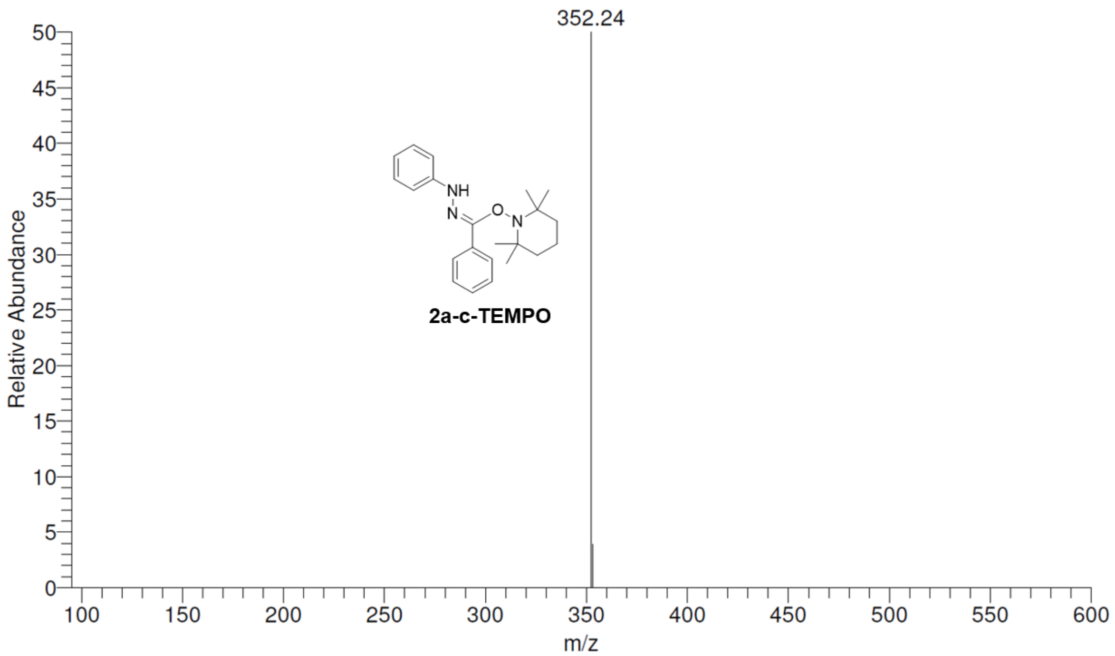

Supplementary Figure 21. LC-MS evidence for **2a-C-TEMPO**.

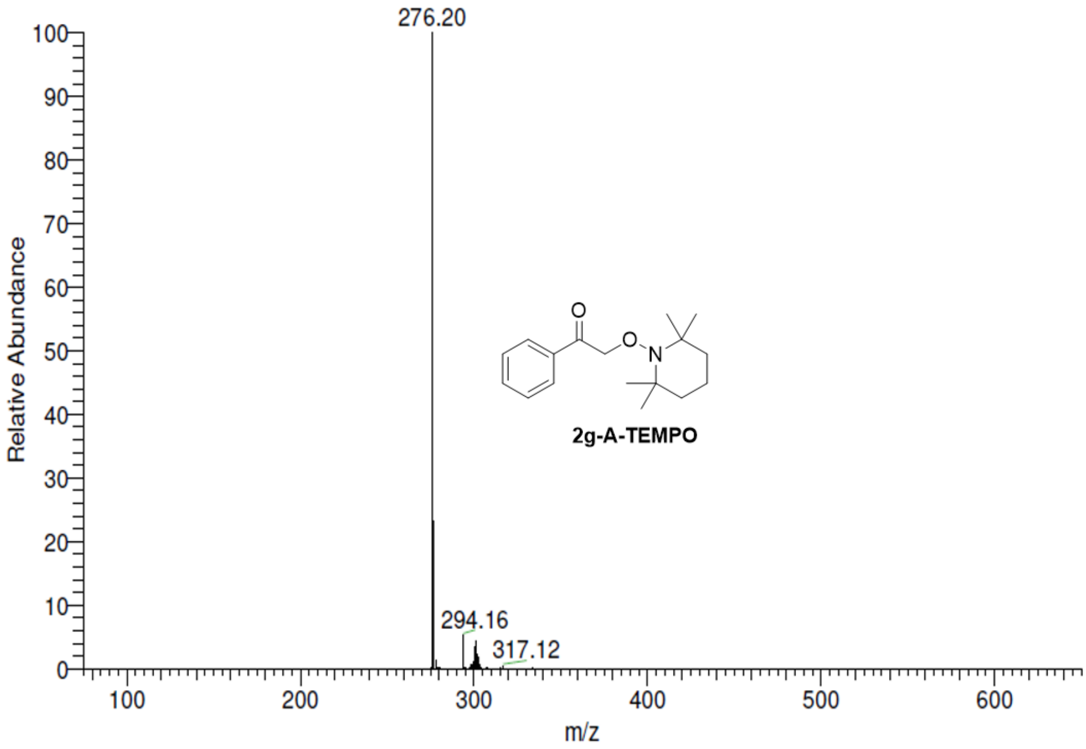

Supplementary Figure 22. LC-MS evidence for **2g-A-TEMPO.**

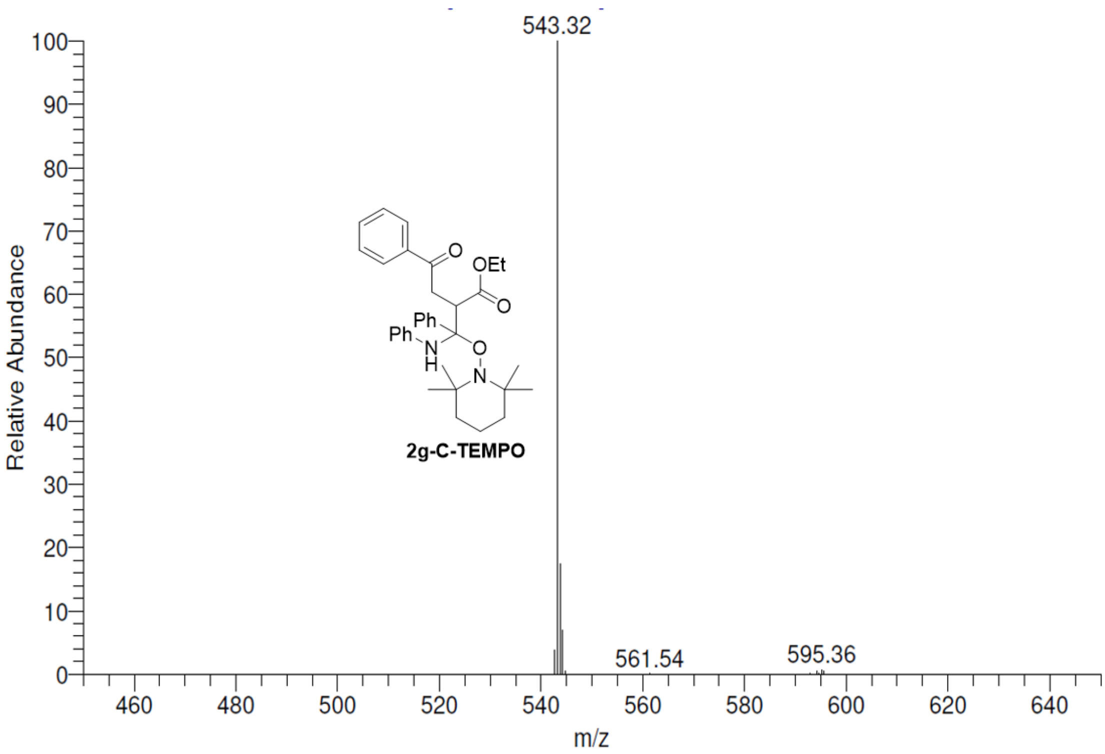

Supplementary Figure 23. LC-MS evidence for the **2g-C-TEMPO**.

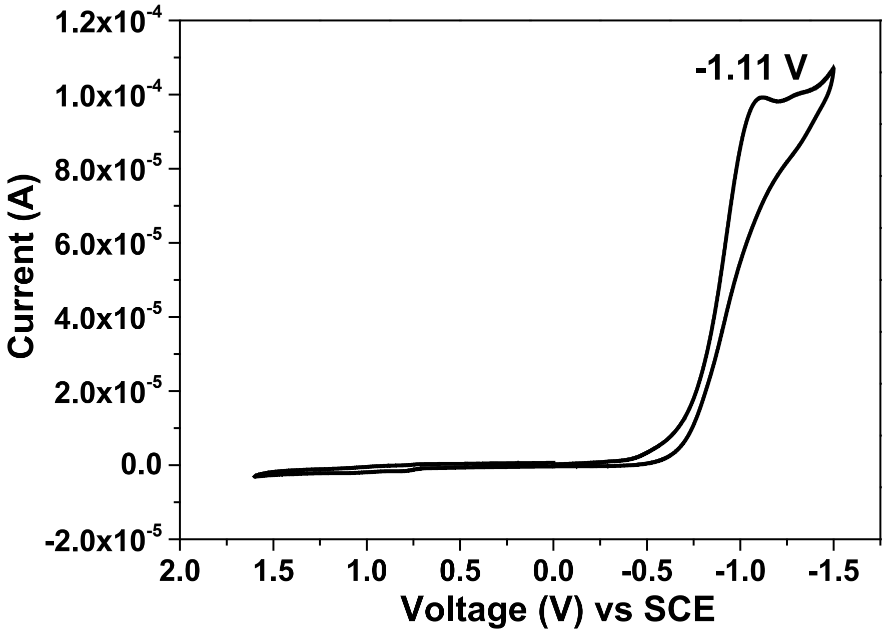

Supplementary Figure 24. CV spectra of 2-Bromoacetophenone using NBu_4PF_6 as electrolyte in degassed CH_3CN. [NBu_4PF_6] = 0.1 M. E_{red} = -1.11 V *vs* SCE. Source data are provided as a Source Data file.

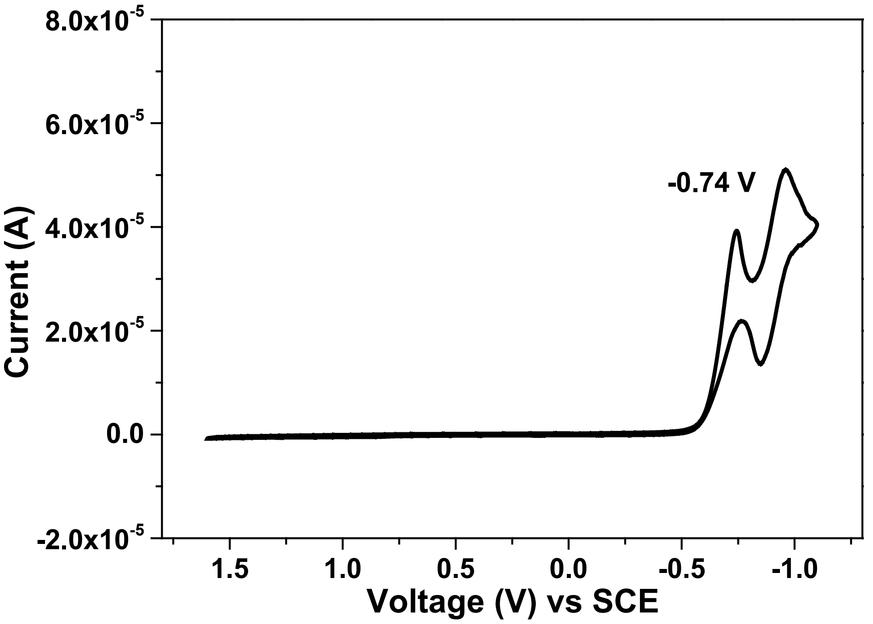

Supplementary Figure 25. CV spectra of 2-bromo-4'-nitroacetophenone using NBu_4PF_6 as electrolyte in degassed CH_3CN. $[NBu_4PF_6]$ = 0.1 M. E_{red} = -0.74 V *vs* SCE. Source data are provided as a Source Data file.

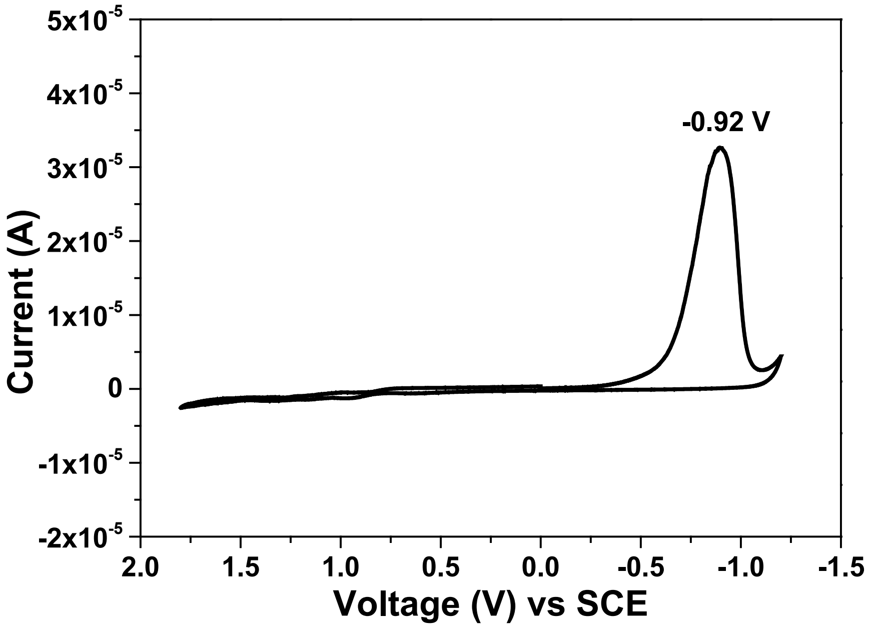

Supplementary Figure 26. CV spectra of 3-(bromoacetyl)coumarin using NBu_4PF_6 as electrolyte in degassed CH_3CN. $[NBu_4PF_6]$ = 0.1 M. E_{red} = -0.92 V *vs* SCE. Source data are provided as a Source Data file.

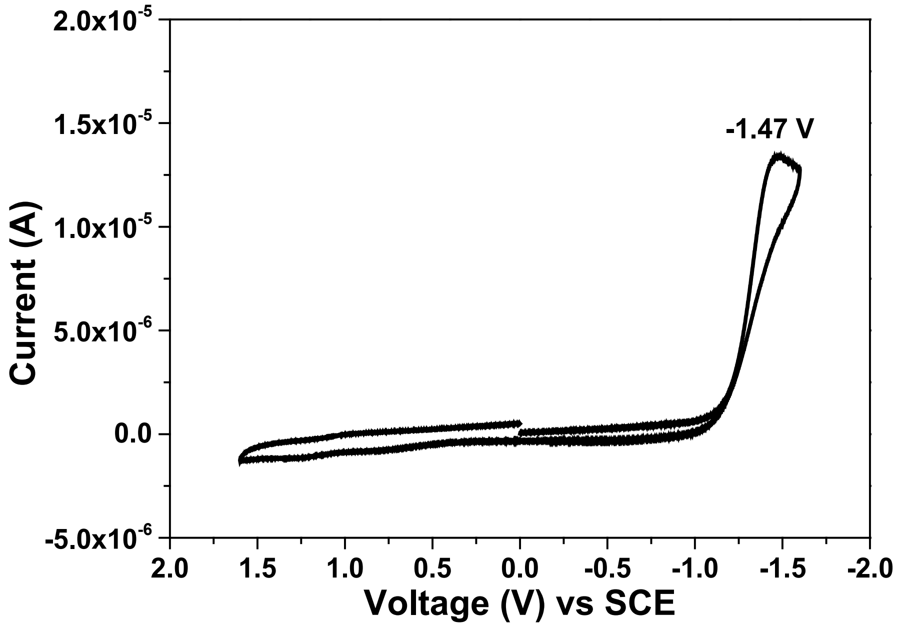

Supplementary Figure 27. CV spectra of 2,4'-dicholoroacetophenone using NBu_4PF_6 as electrolyte in degassed CH_3CN. $[NBu_4PF_6]$ = 0.1 M. E_{red} = -1.47 V *vs* SCE. Source data are provided as a Source Data file.

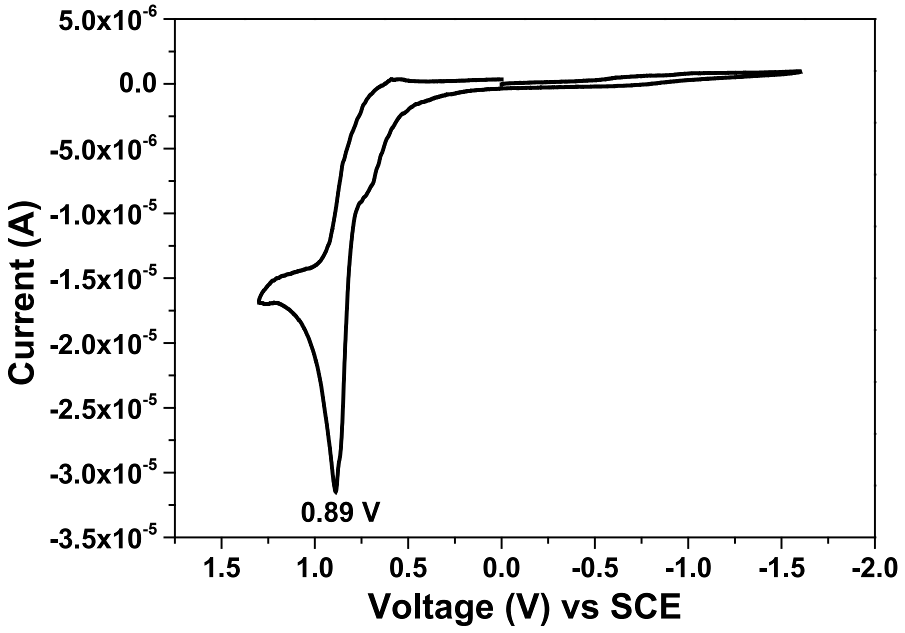

Supplementary Figure 28. CV spectra of 4-phenylmorpholine using NBu_4PF_6 as electrolyte in degassed CH_3CN. [NBu_4PF_6] = 0.1 M. E_{ox} = 0.89 V *vs* SCE. Source data are provided as a Source Data file.

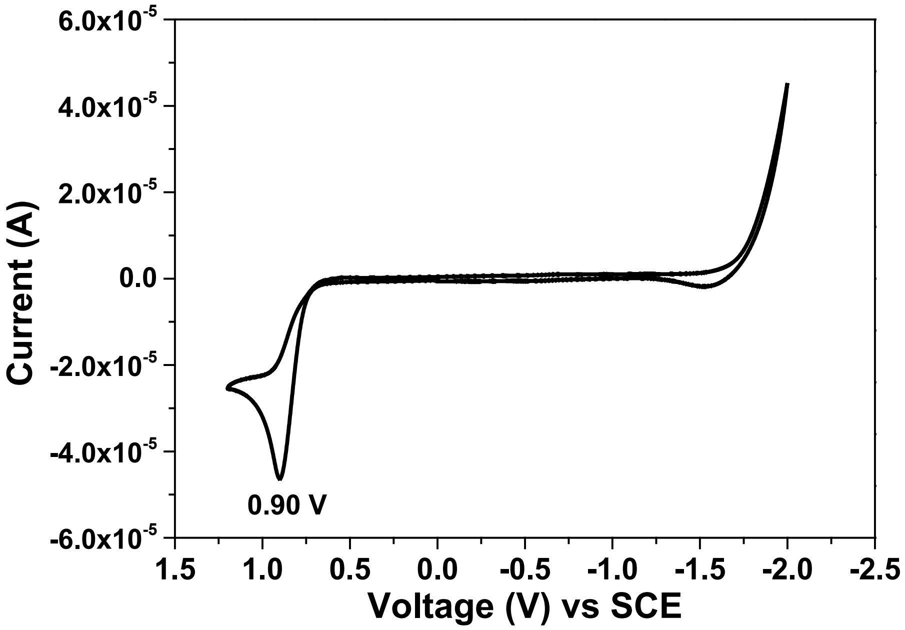

Supplementary Figure 29. CV spectra of 2-phenyl-1,2,3,4-tetrahydroisoquinoline using NBu_4PF_6 as electrolyte in degassed CH_3CN. [NBu_4PF_6] = 0.1 M. E_{ox} = 0.90 V *vs* SCE. Source data are provided as a Source Data file.

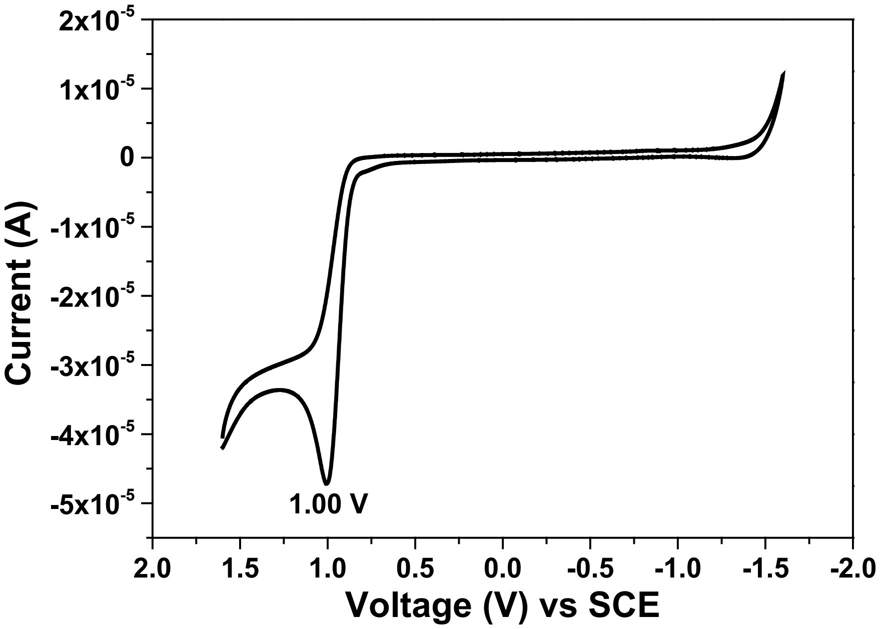

Supplementary Figure 30. CV spectra of (*E*)-1-benzylidene-2-phenylhydrazine using NBu_4PF_6 as electrolyte in degassed CH_3CN. [NBu_4PF_6] = 0.1 M. E_{ox}= 1.00 V *vs* SCE. Source data are provided as a Source Data file.

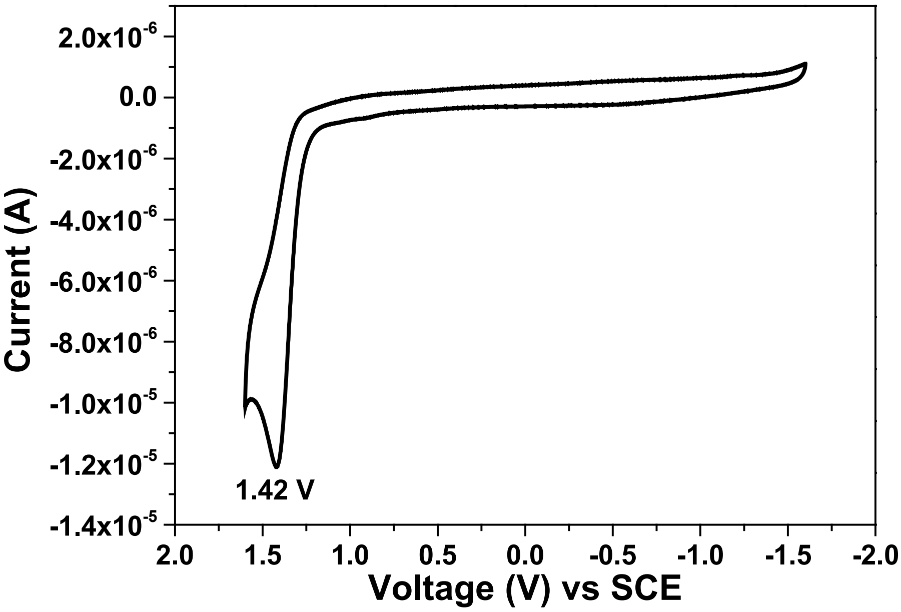

Supplementary Figure 31. CV spectra of ethyl (*E*)-3-phenyl-3-(phenylamino)acrylate using NBu_4PF_6 as electrolyte in degassed CH_3CN. [NBu_4PF_6] = 0.1 M. E_{ox}= 1.42 V *vs* SCE. Source data are provided as a Source Data file.

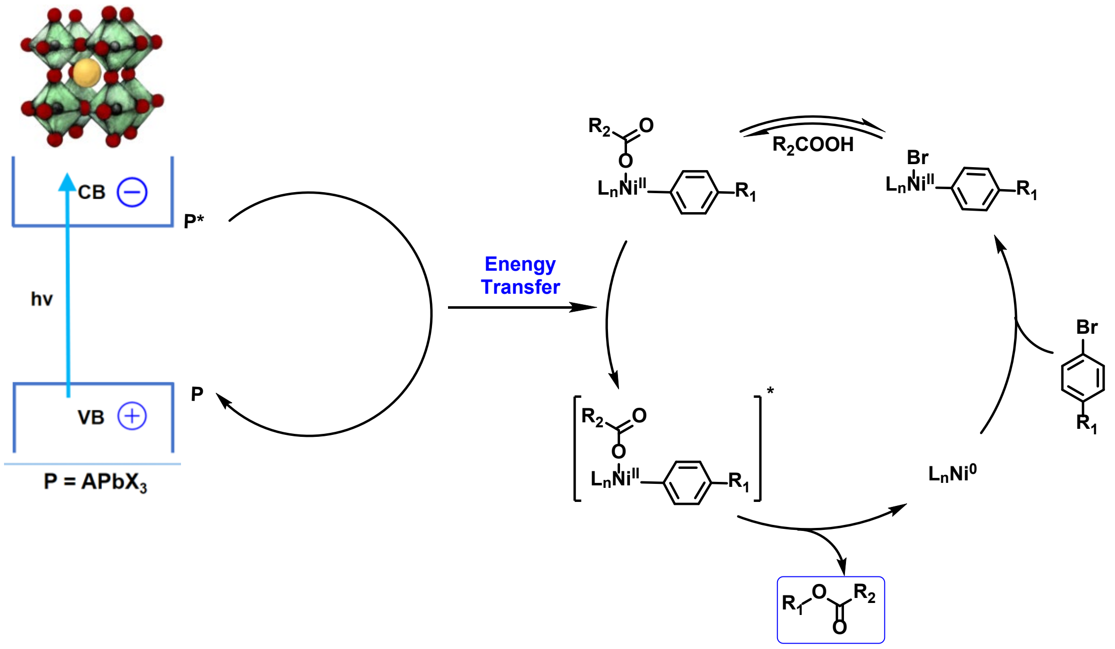

Supplementary Figure 32. Proposed mechanism for the C-O bond formation that was similar to previously explored photocatalytic C-O formation reaction by Macmillan *et al.*[1]

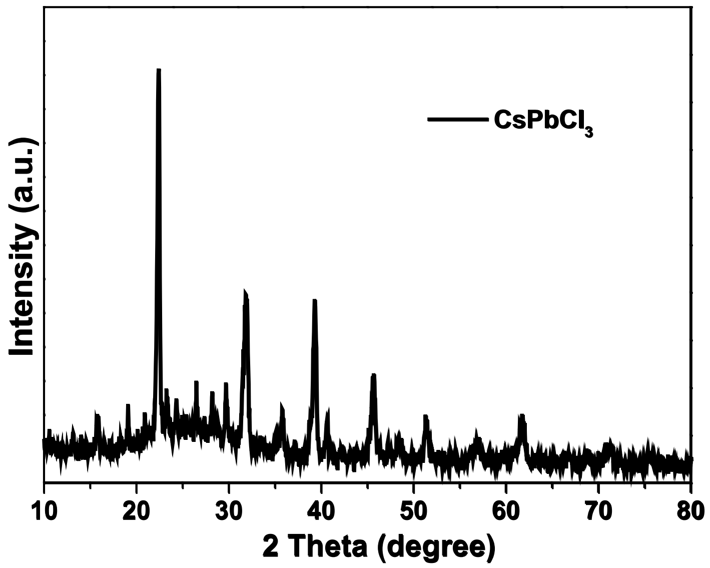

Supplementary Figure 33. XRD for the $CsPbCl_3$ forms from the **2j** reaction after irradiation for 24 h. Source data are provided as a Source Data file.

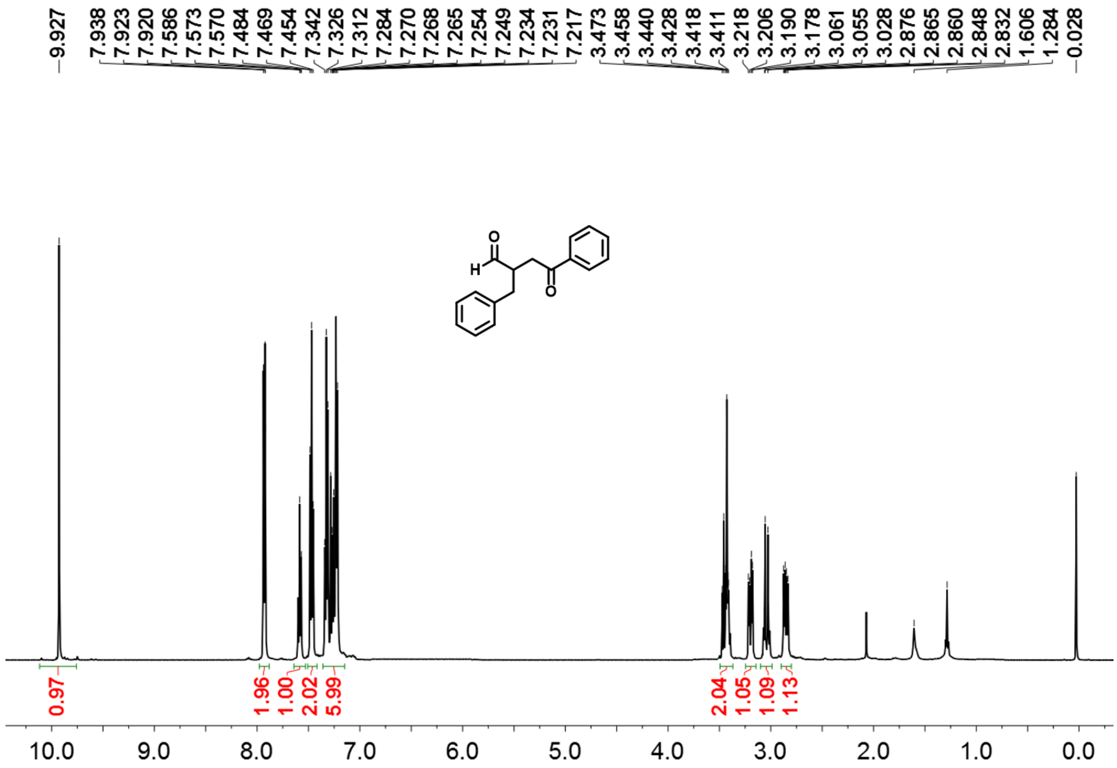

Supplementary Figure 34. ^{1}H NMR spectrum of **1a**.

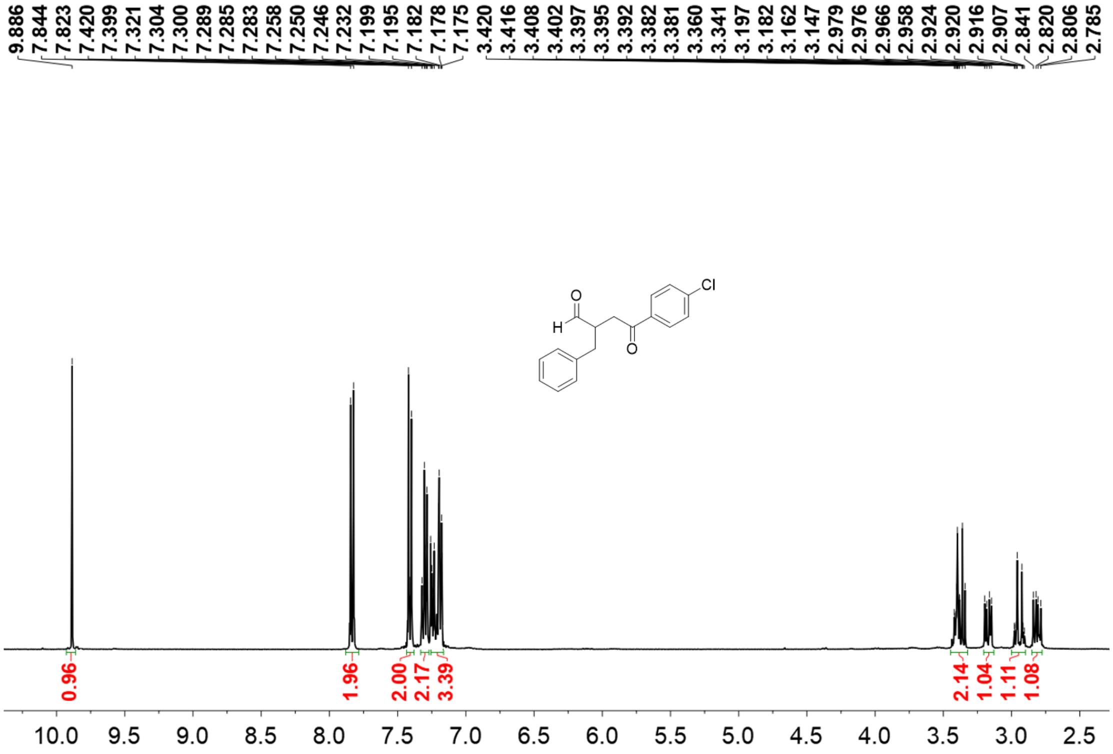

Supplementary Figure 35. ^{1}H NMR spectrum of **1b**.

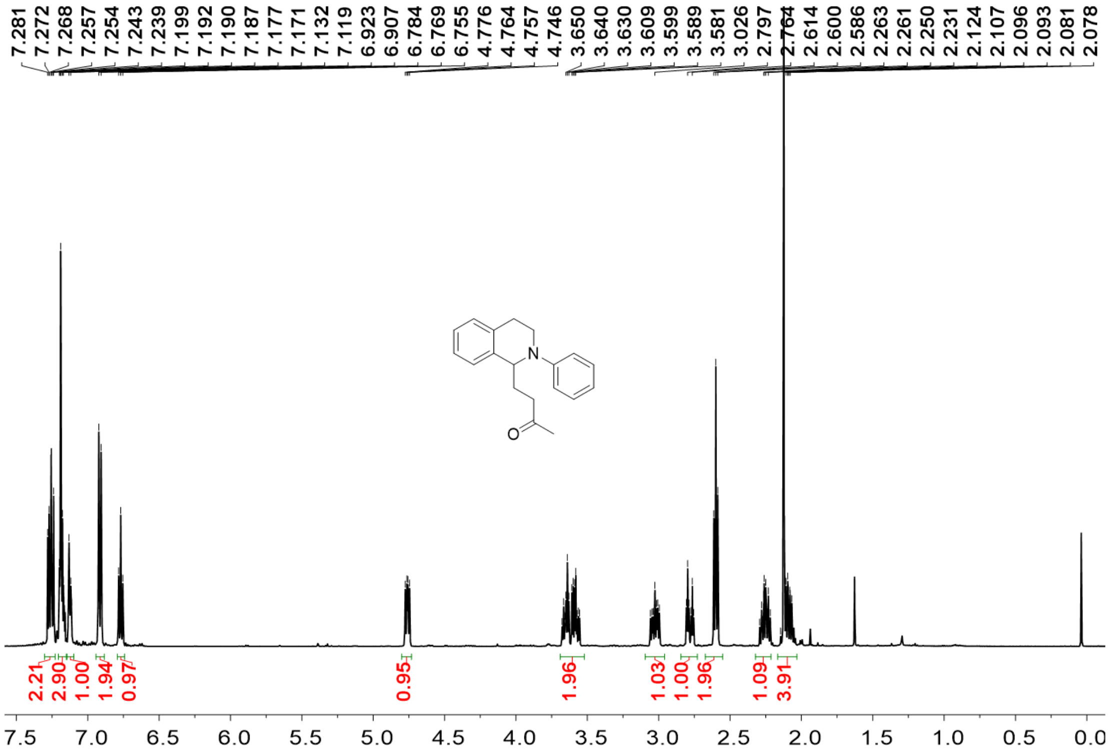

Supplementary Figure 36. ^{1}H NMR spectrum of **1c**.

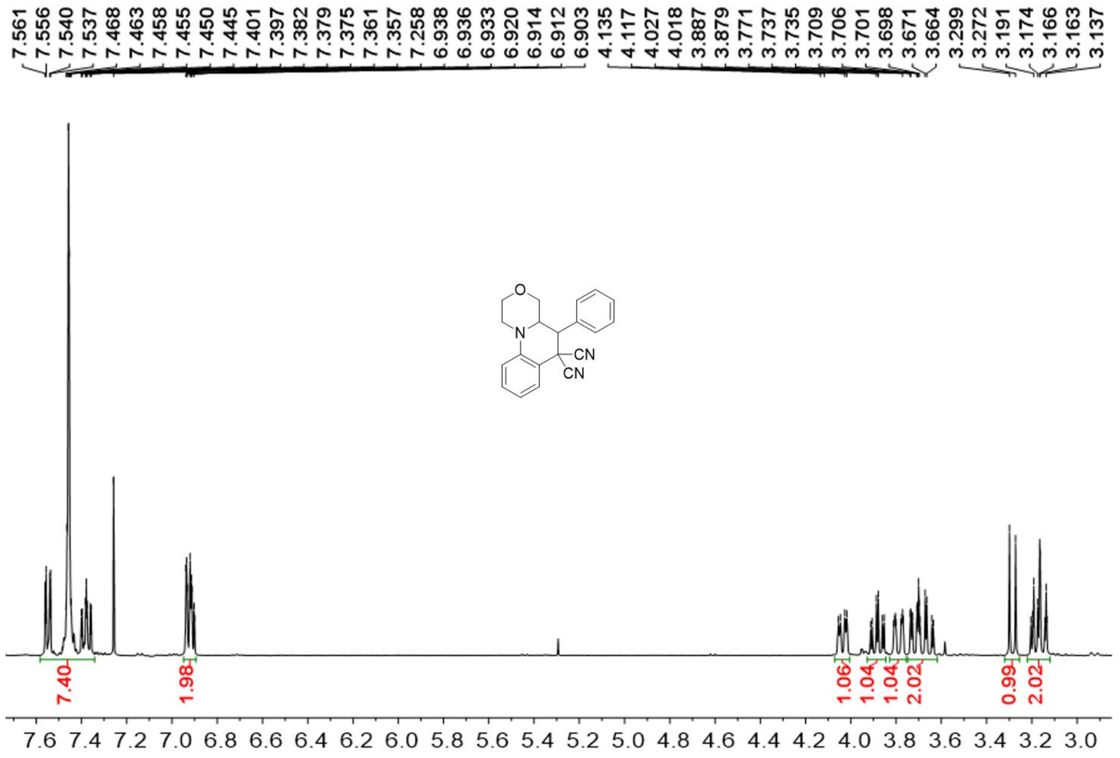

Supplementary Figure 37. ^{1}H NMR spectrum of **1d**.

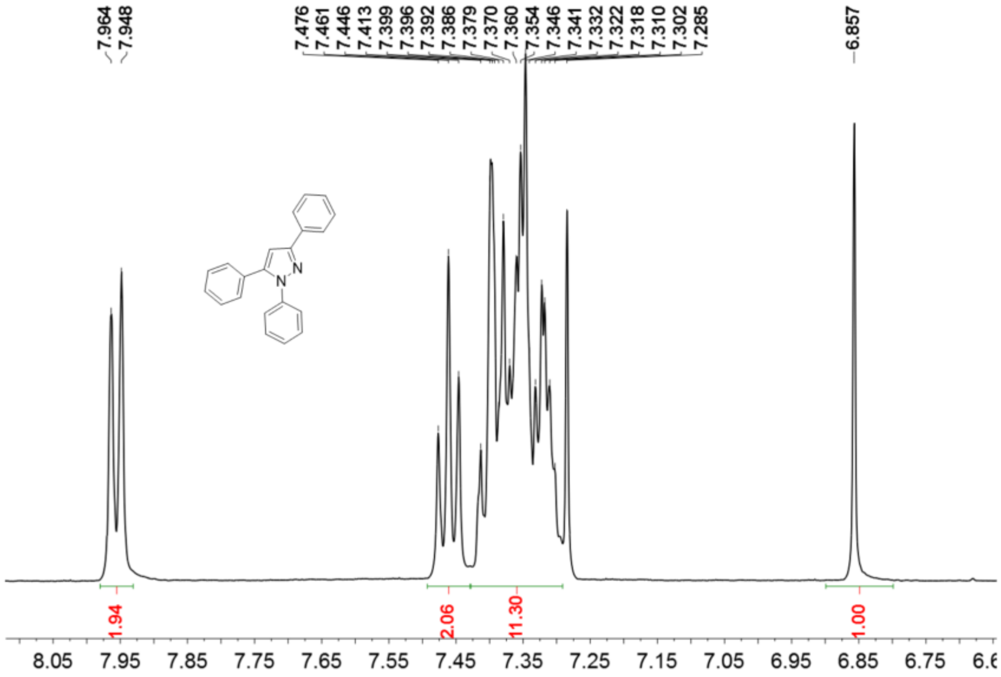

Supplementary Figure 38. ^{1}H NMR spectrum of **2a**.

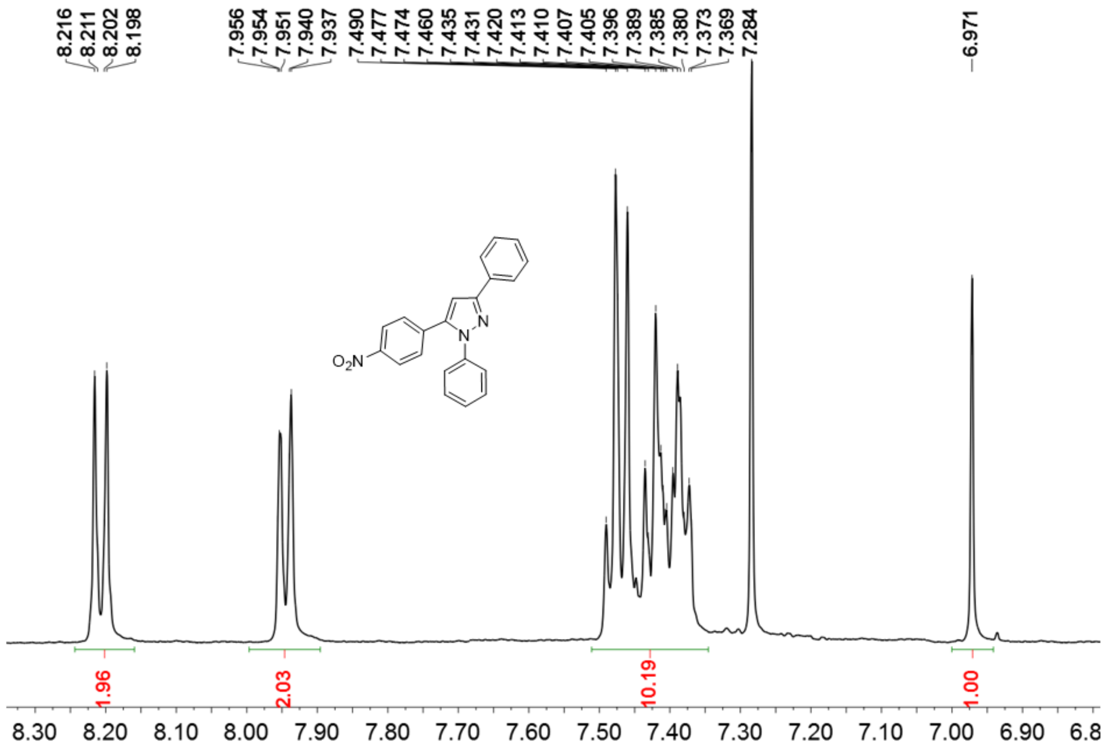

Supplementary Figure 39. ^{1}H NMR spectrum of **2b**.

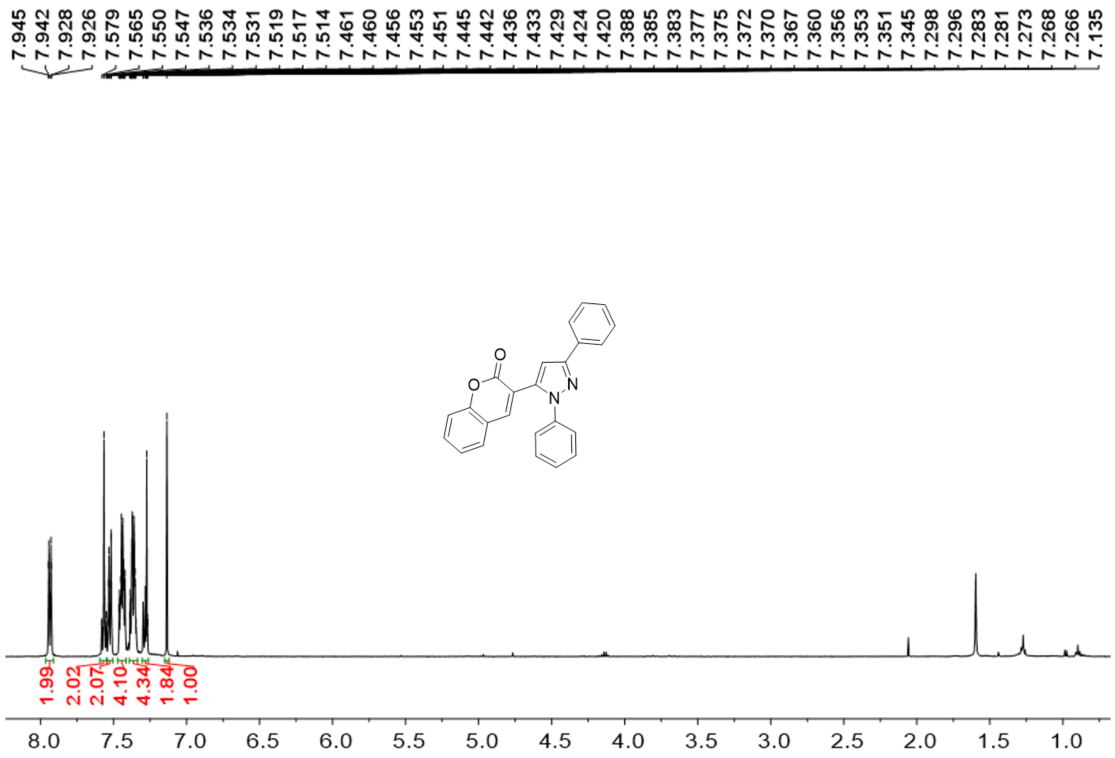

Supplementary Figure 40. ^{1}H NMR spectrum of **2c**.

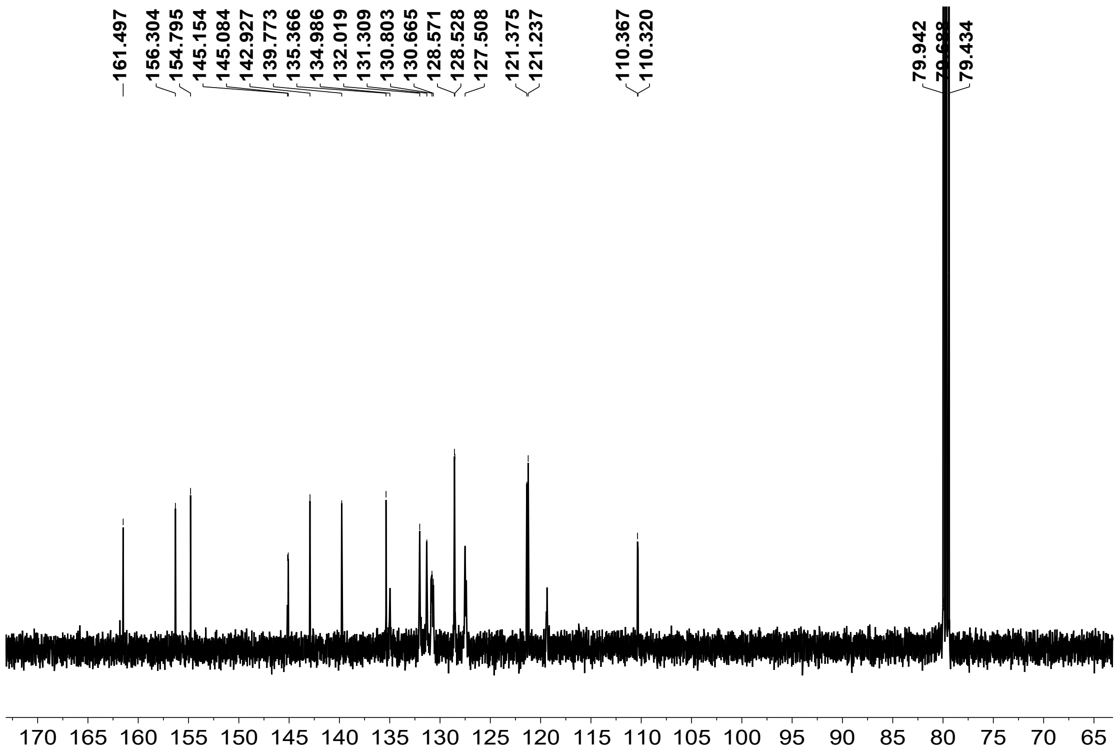

Supplementary Figure 41. ^{13}C NMR spectrum of **2c**.

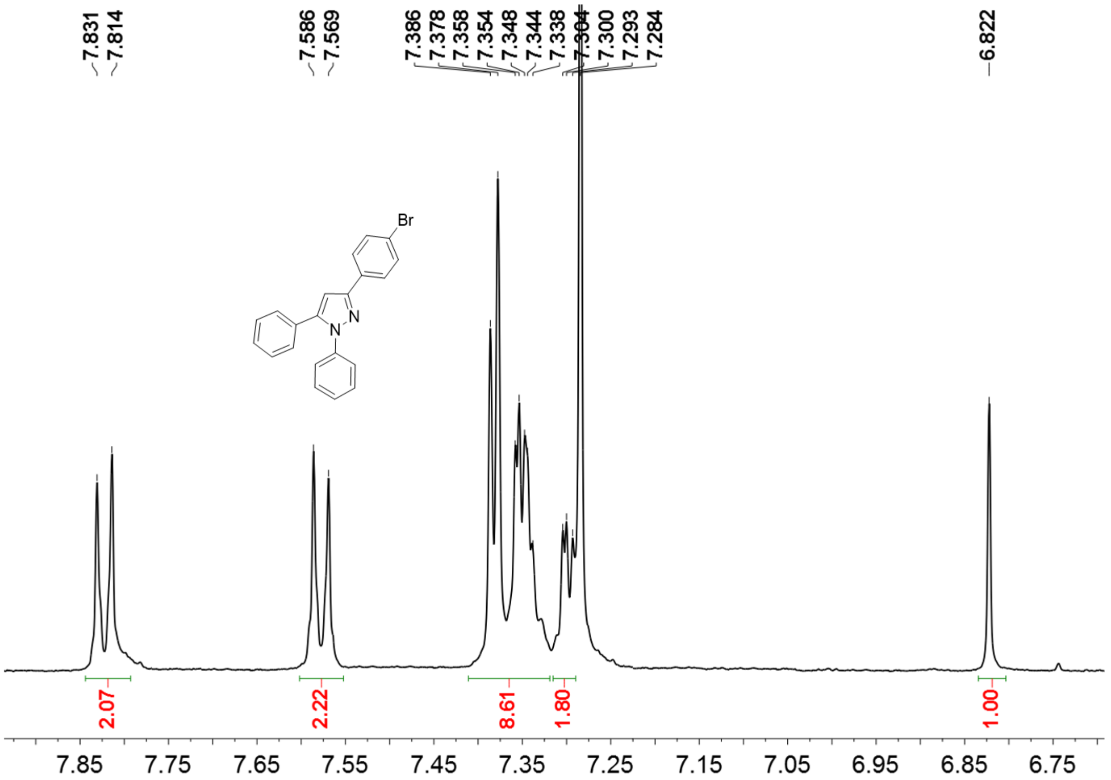

Supplementary Figure 42. ^{1}H NMR spectrum of **2d**.

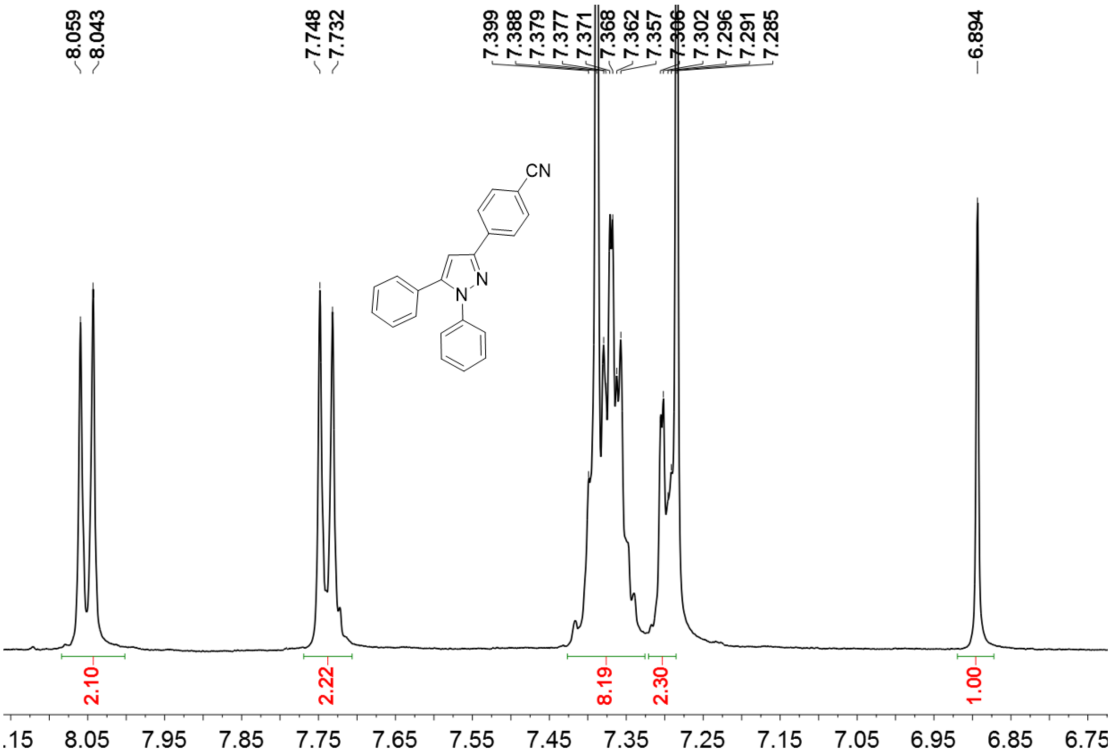

Supplementary Figure 43. ^{1}H NMR spectrum of **2e**.

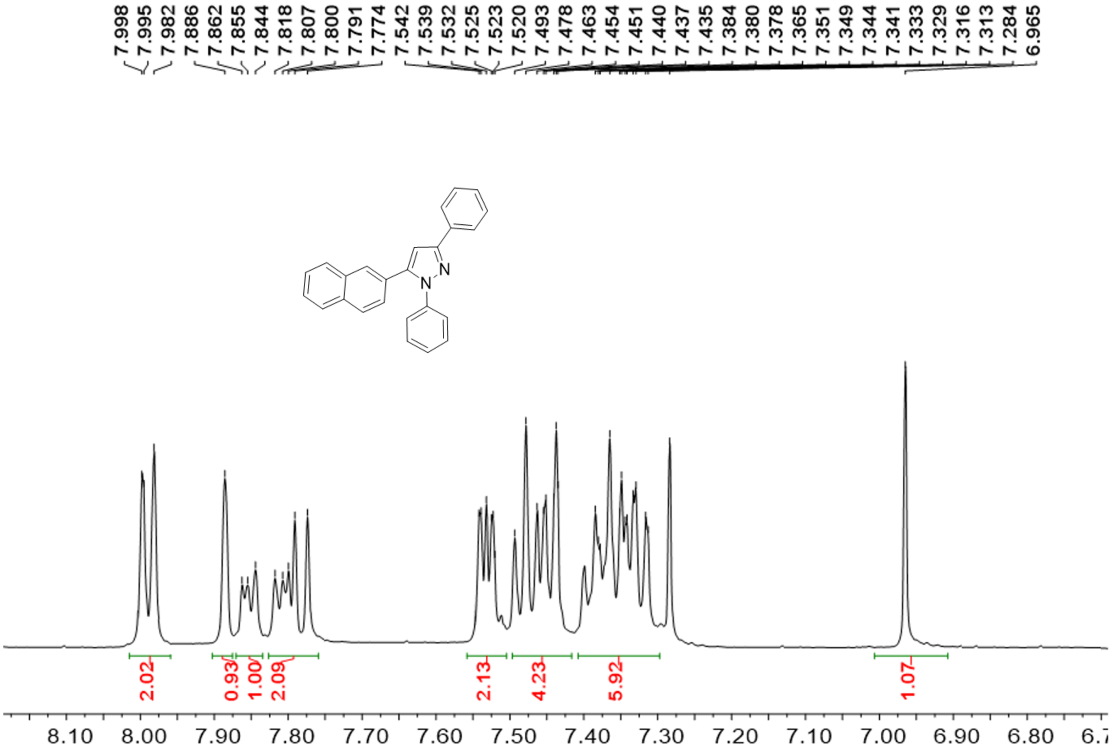

Supplementary Figure 44. ^{1}H NMR spectrum of **2f**.

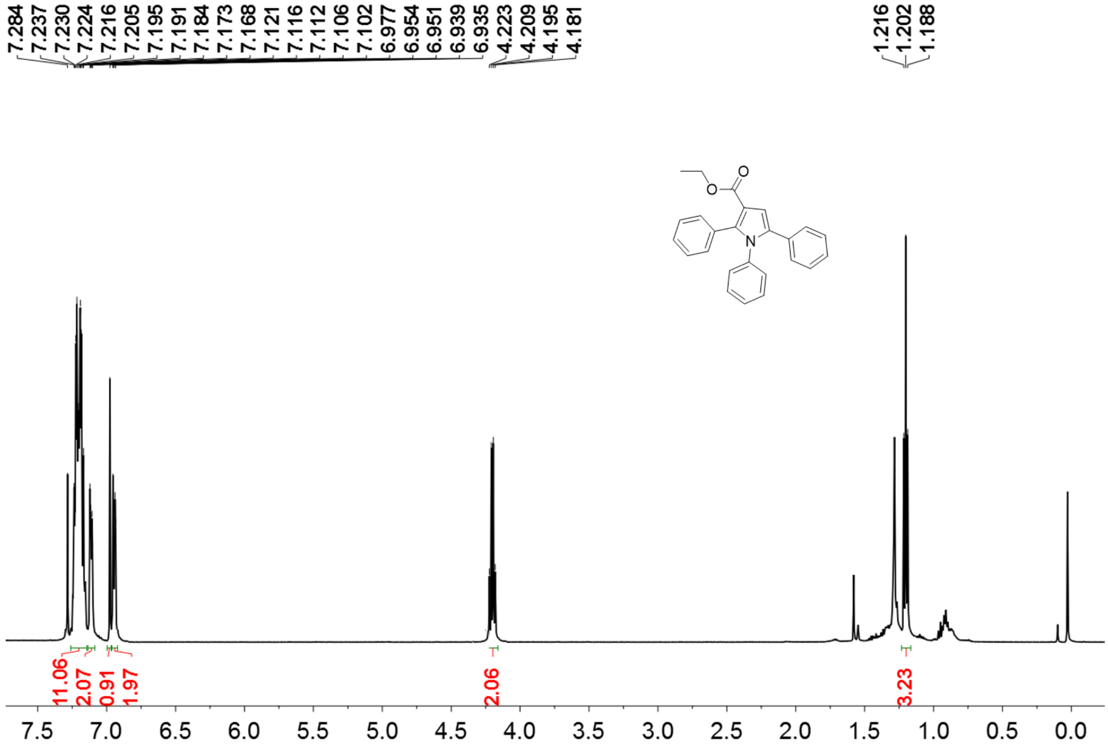

Supplementary Figure 45. ^{1}H NMR spectrum of **2g.**

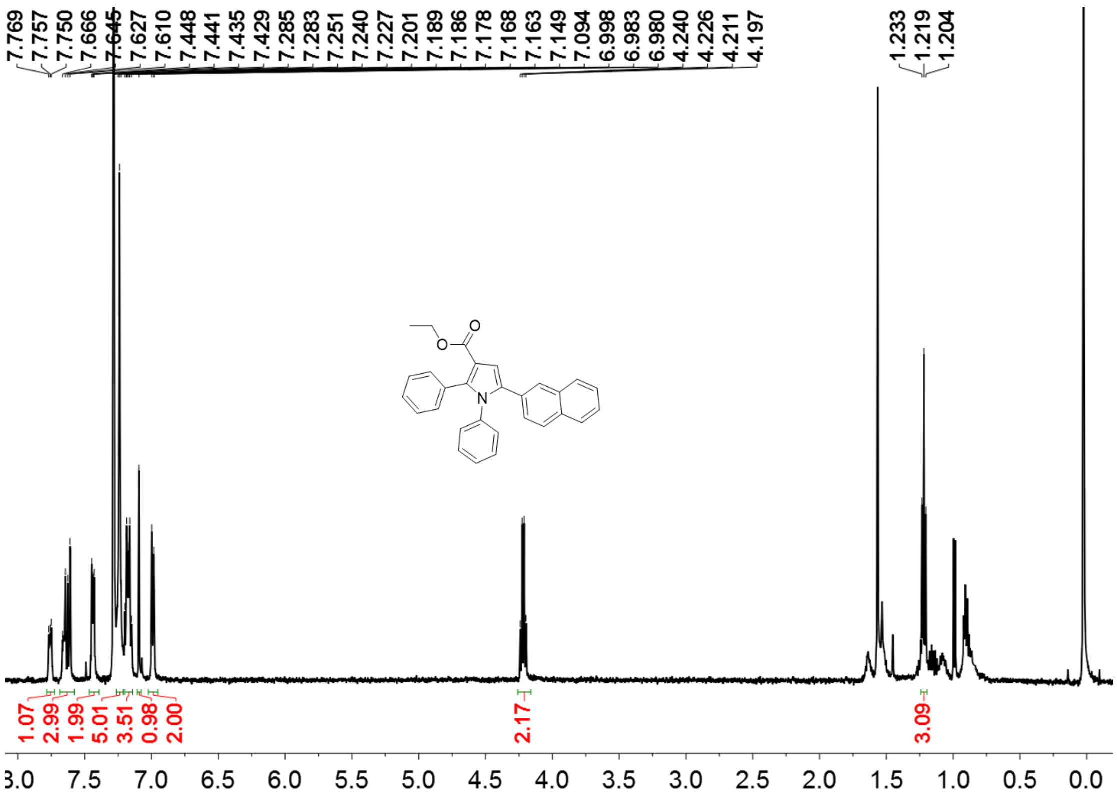

Supplementary Figure 46. ^{1}H NMR spectrum of **2h.**

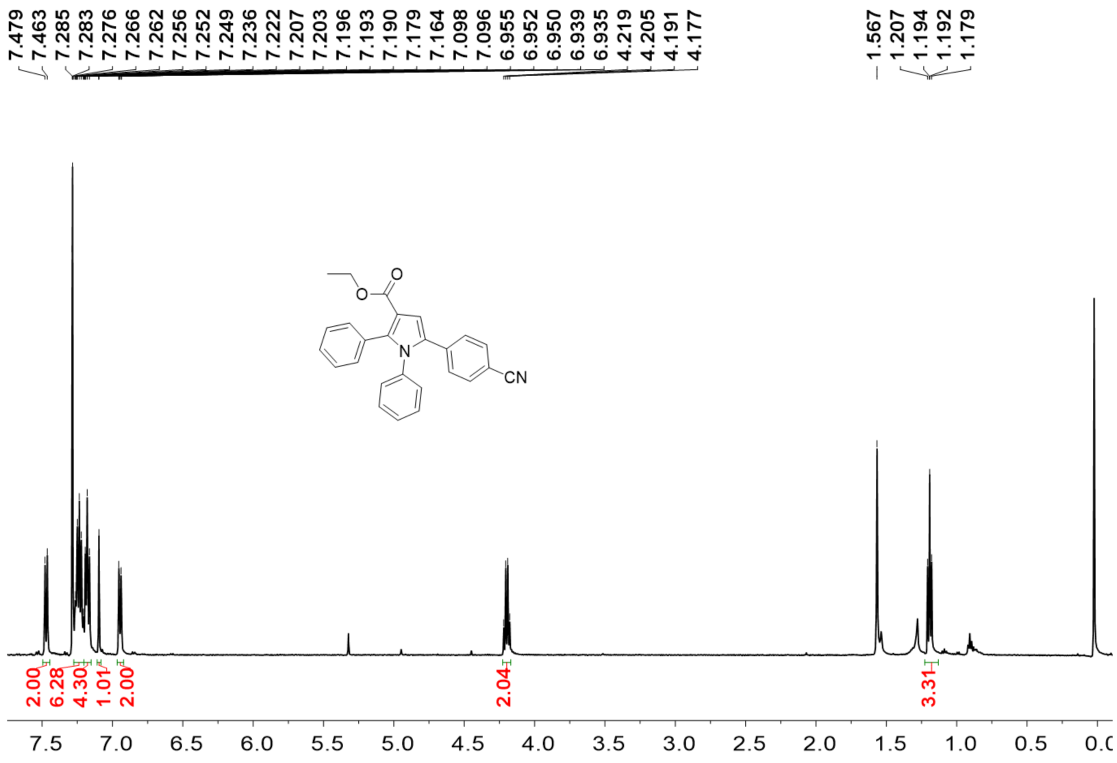

Supplementary Figure 47. ^{1}H NMR spectrum of **2i**.

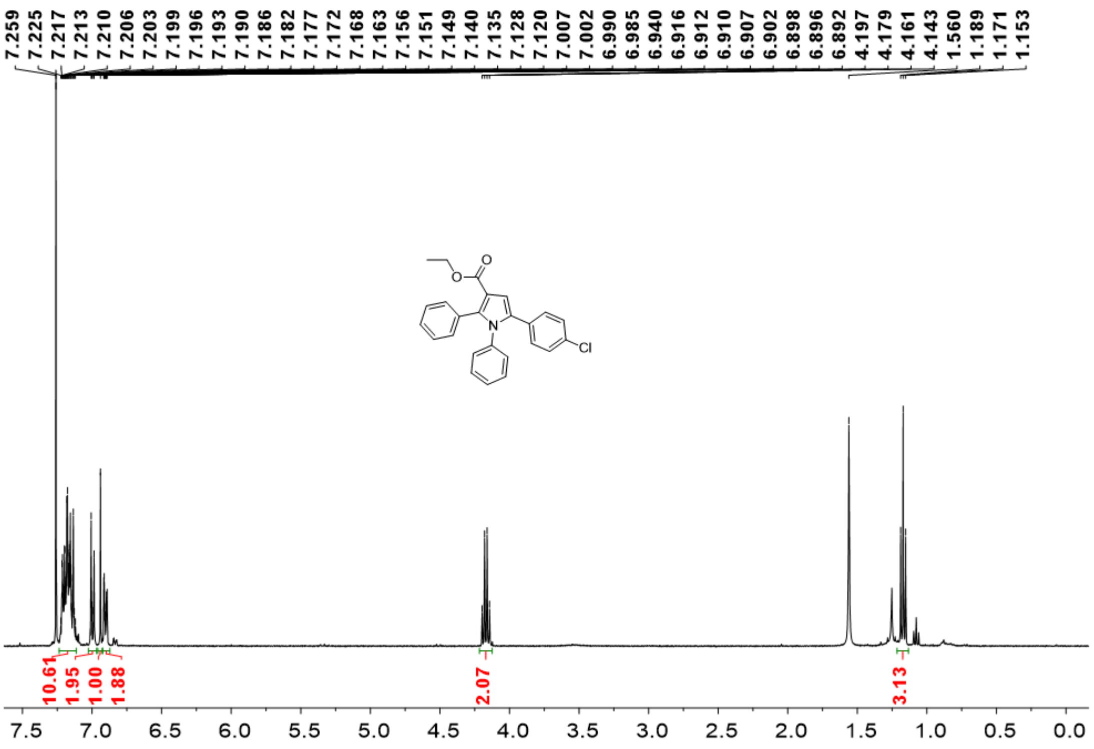

Supplementary Figure 48. ^{1}H NMR spectrum of **2j**.

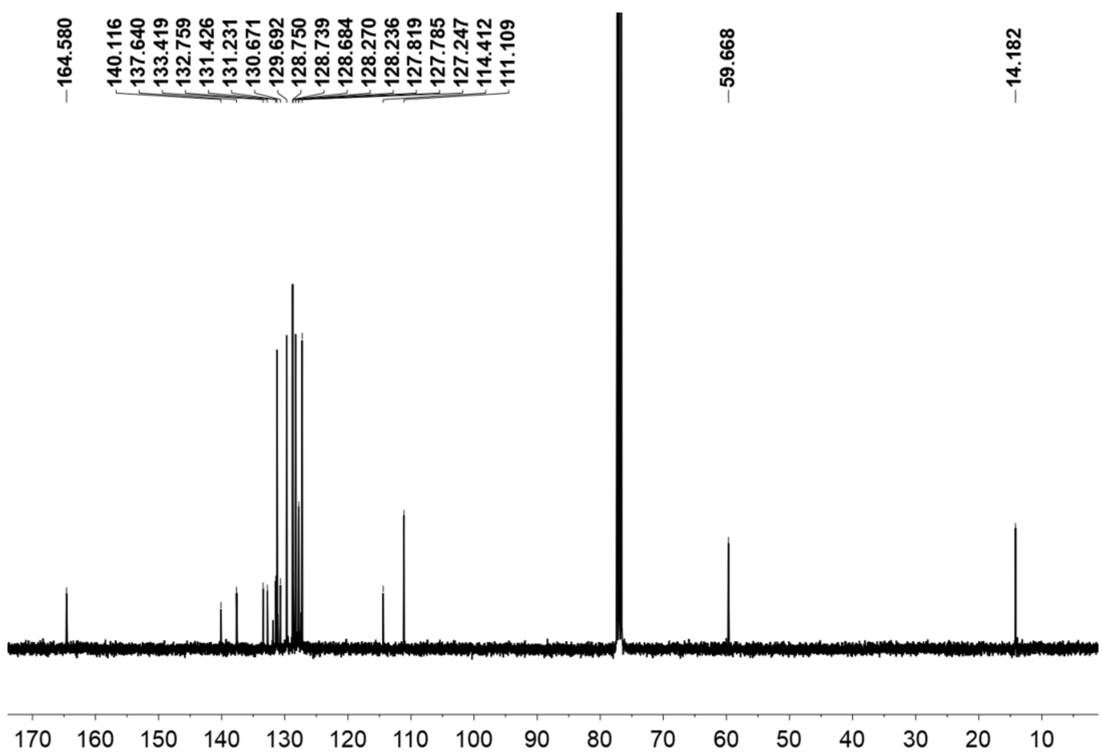

Supplementary Figure 49. ^{13}C NMR spectrum of **2j**.

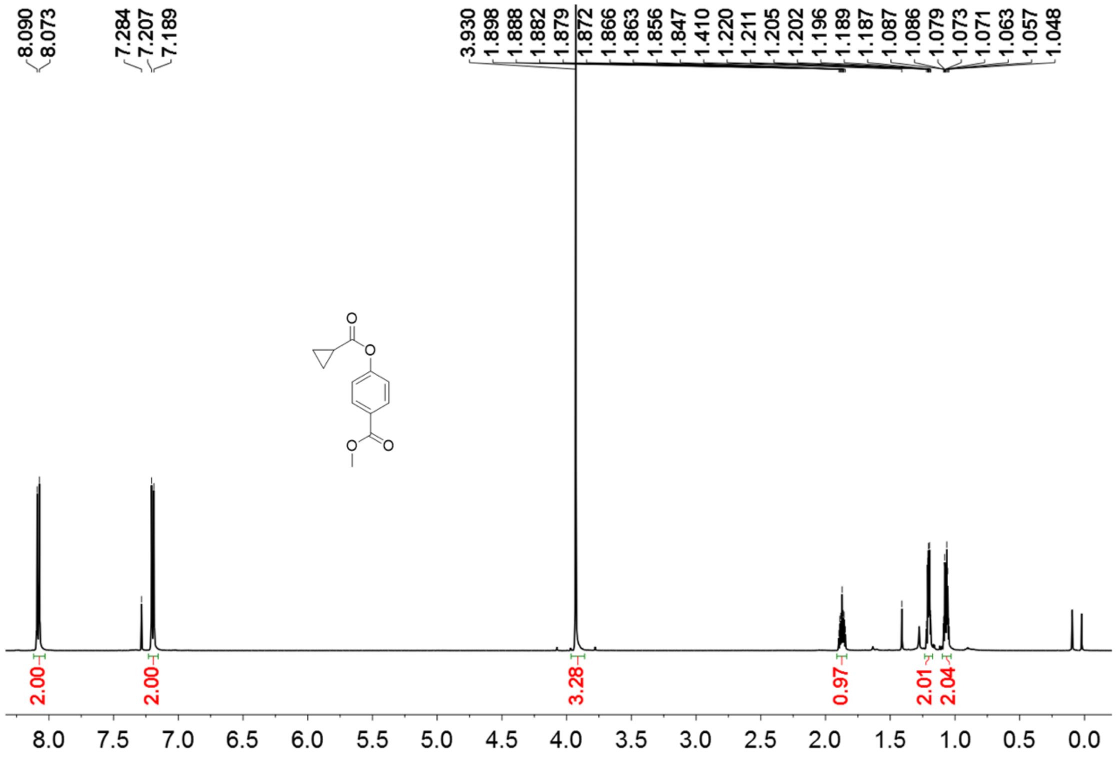

Supplementary Figure 50. ^{1}H NMR spectrum of **3a**.

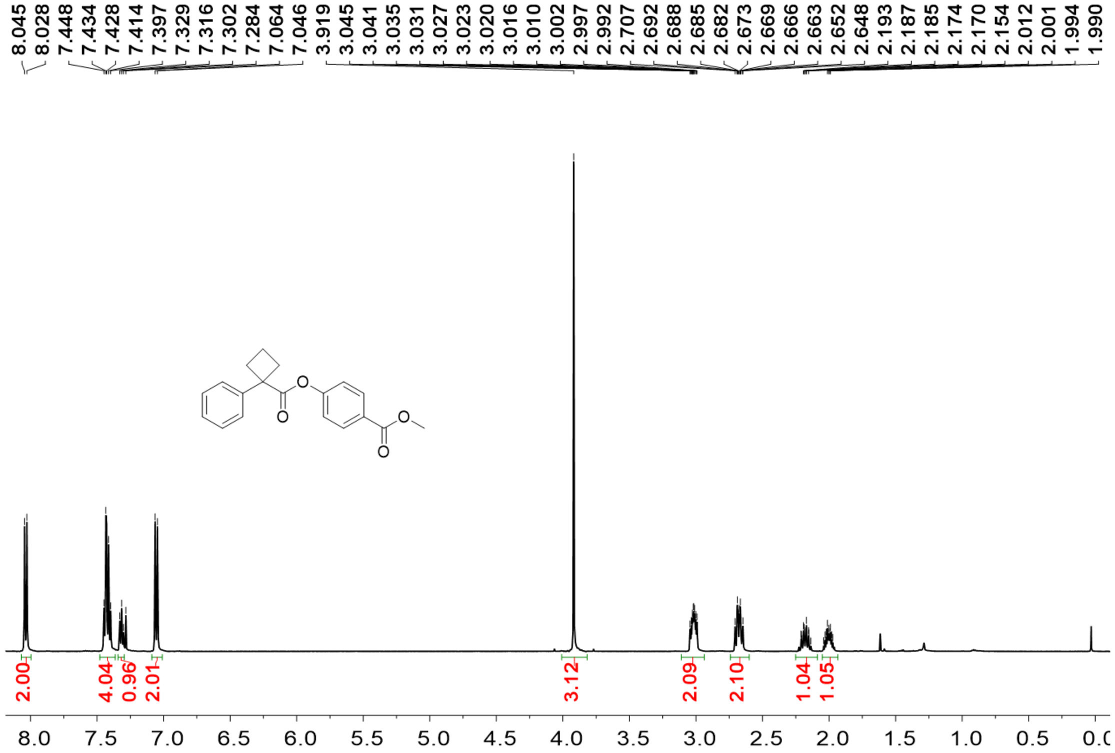

Supplementary Figure 51. ^{1}H NMR spectrum of **3b**.

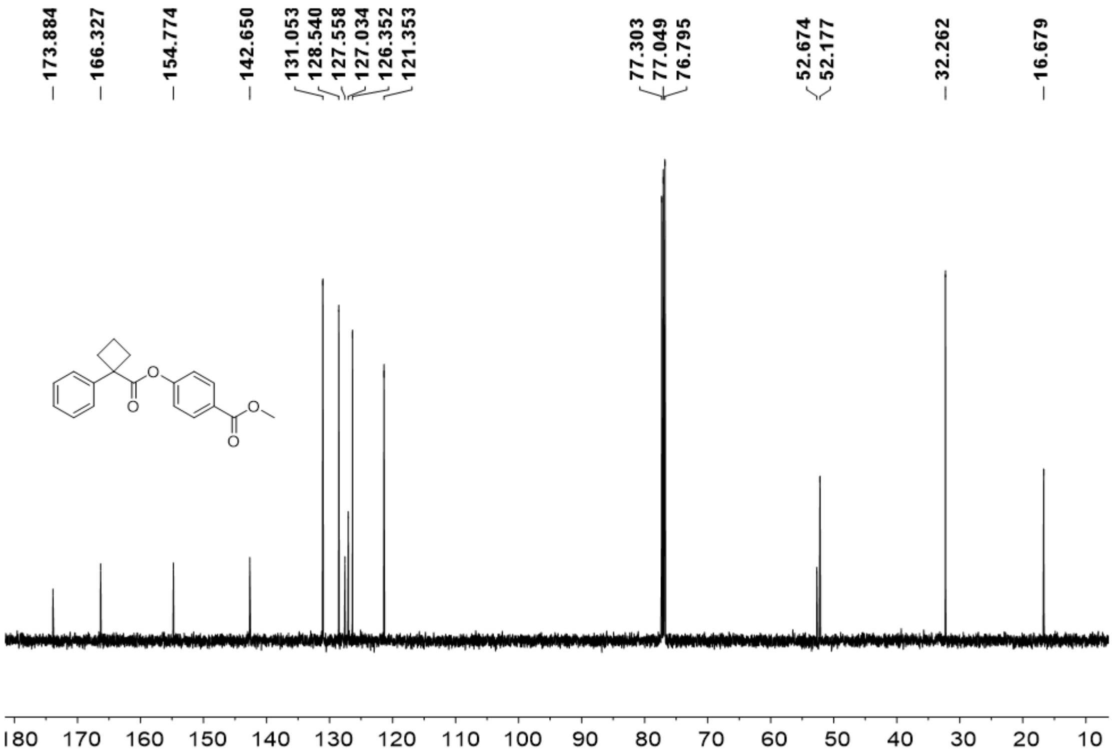

Supplementary Figure 52. ^{13}C NMR spectrum of **3b**.

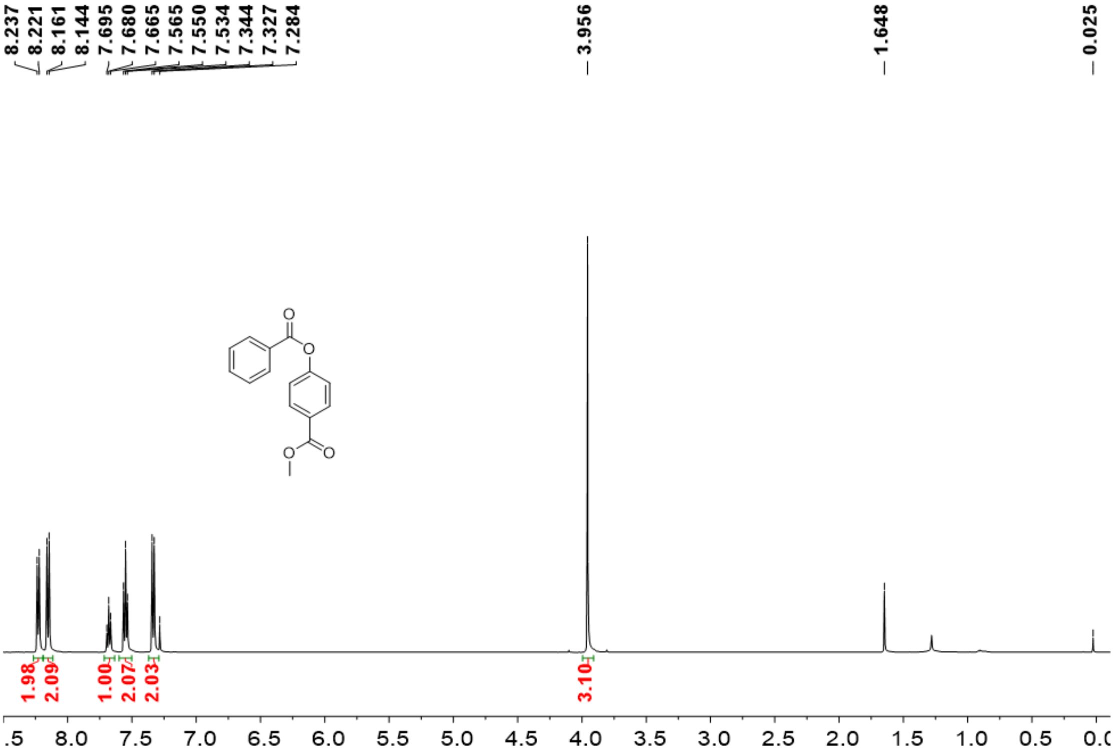

Supplementary Figure 53. ^{1}H NMR spectrum of **3c**.

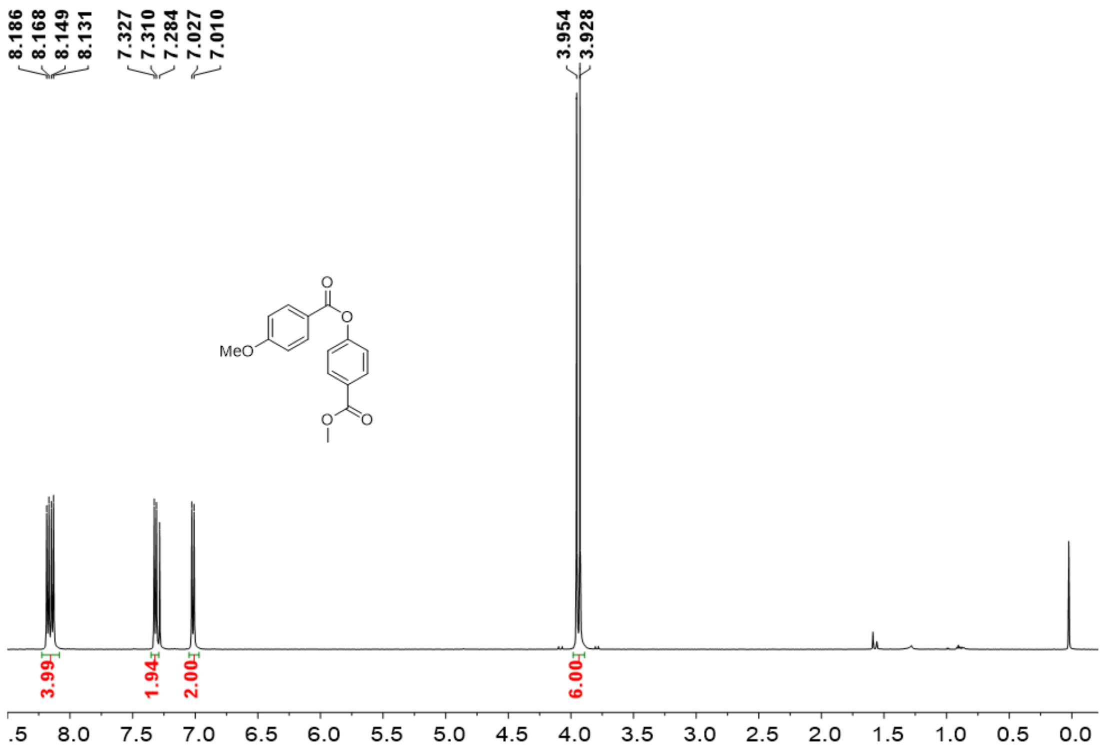

Supplementary Figure 54. ^{1}H NMR spectrum of **3d.**

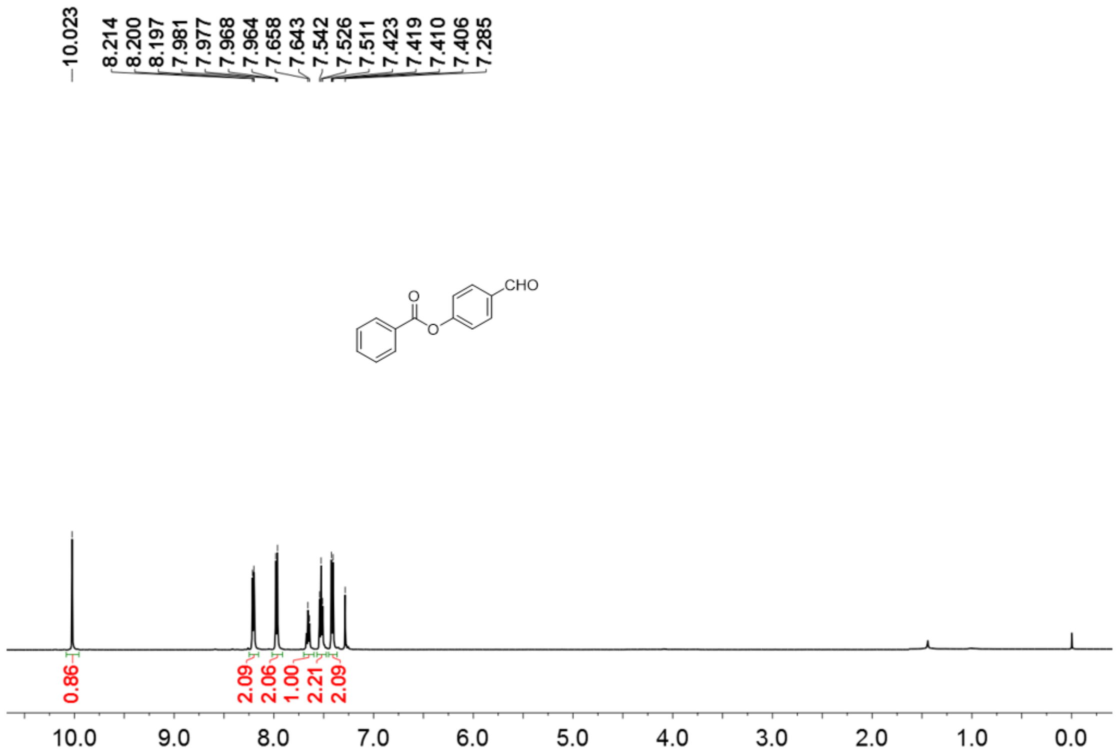

Supplementary Figure 55. ^{1}H NMR spectrum of **3e**.

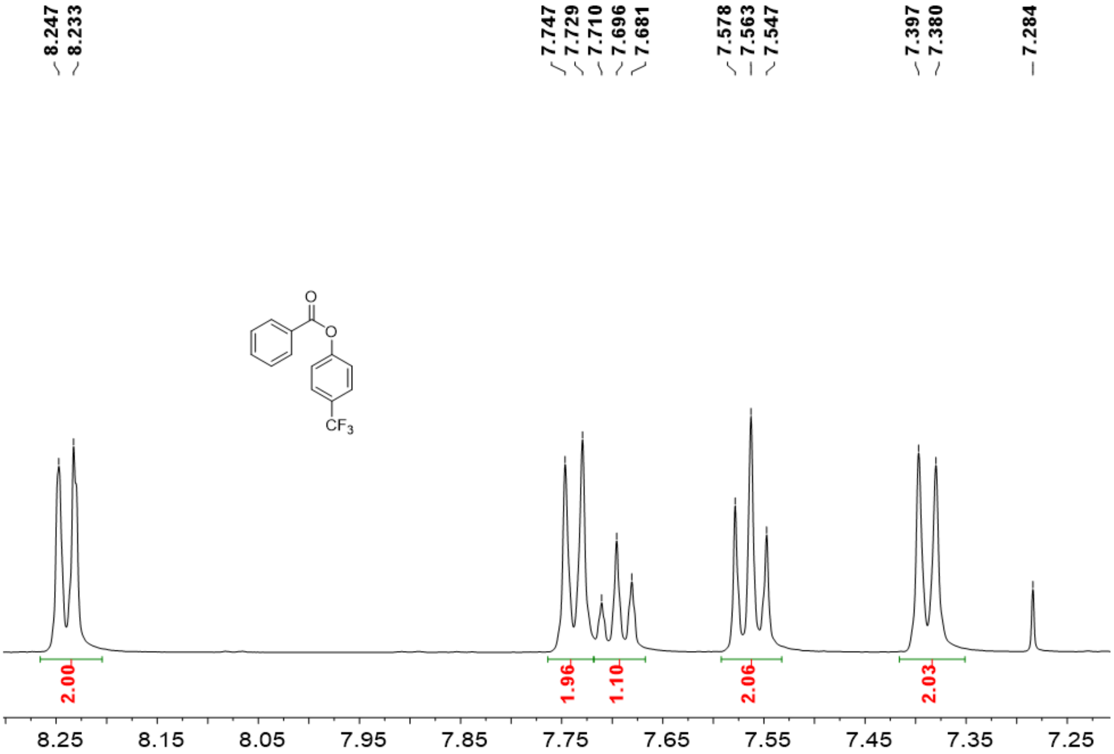

Supplementary Figure 56. ^{1}H NMR spectrum of **3f**.

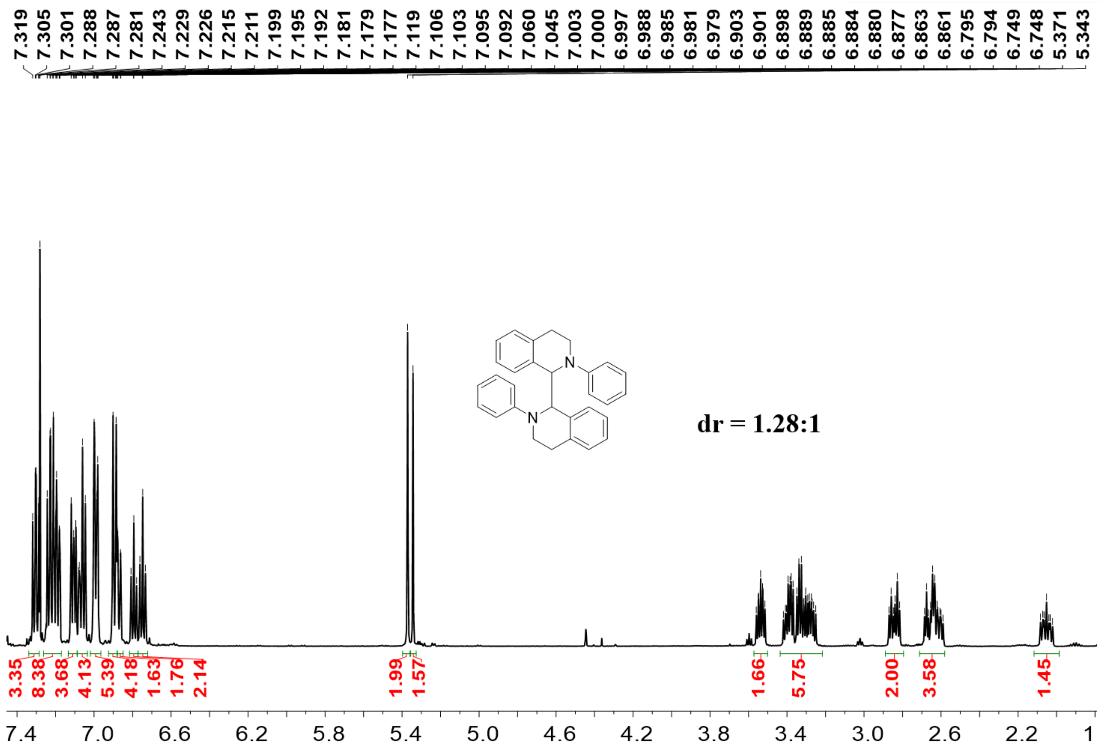

Supplementary Figure 57. ^{1}H NMR spectrum of **1c-C**.

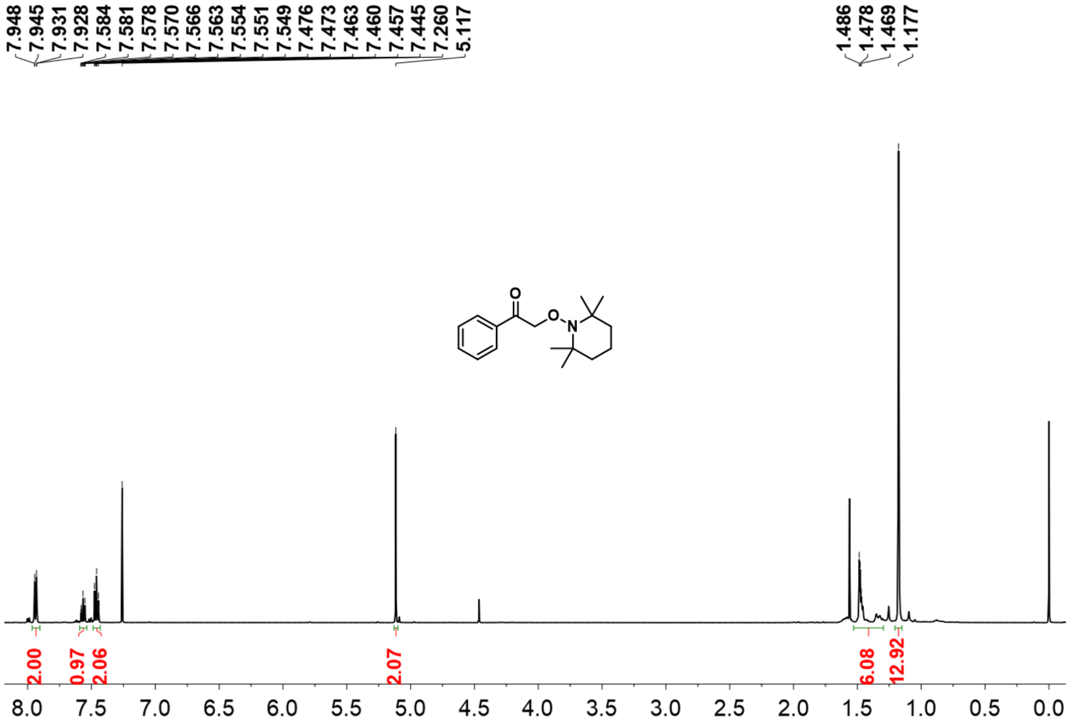

Supplementary Figure 58. The ^{1}H NMR for **2a-A-TEMPO**.

Supplementary Table 1. Comparison of $CsPbBr_3$ **P1** and other photocatalysts for the synthesis of **1a**.[a]

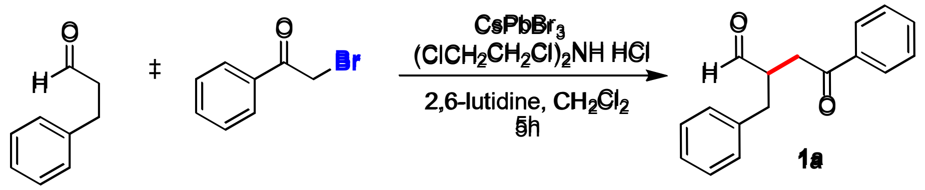

Entry	Photocatalyst	Variation of conditions	Condition	Yield (%) [b]
1	$CsPbBr_3$		N_2	85
2	$CsPbBr_3$		air	84
3	$CsPbBr_3$	0.5 mg	air	71
4	$CsPbBr_3$	2.0 mg	air	85
5	525 nm CdSe QDs	Hexane suspention	N_2	15
6	525 nm CdSe QDs	Hexane suspention	air	Trace
7	525 nm CdSe QDs	Hexane suspention with oleic acid	N_2	53
8	525 nm CdSe QDs	Hexane suspention with oleic acid	air	<5
9	550 nm CdSe QDs	Hexane suspention	N_2	13
10	550 nm CdSe QDs	Hexane suspention	air	Trace
11	550 nm CdSe QDs	Hexane suspention with oleic acid	N_2	31
12	550 nm CdSe QDs	Hexane suspention with oleic acid	air	Trace
13	525 nm CdSe QDs	isolated powder	N_2	Trace
14	525 nm CdSe QDs	isolated powder	air	Trace
15	550 nm CdSe QDs	isolated powder	N_2	Trace
16	550 nm CdSe QDs	isolated powder	air	Trace
17	$Ir(ppy)_3$		N_2	83
18	$Ir(ppy)_3$		air	79
19	$Ru(bpy)_3(PF_6)_2$		N_2	83
20	$Ru(bpy)_3(PF_6)_2$		air	<5
21	DMPDP		N_2	81
22	DMPDP		air	18
23	TiO_2 32 nm		N_2	<5
24	TiO_2 32 nm		air	<5
25	$PbBr_2$		air	N.R.
26	$PbBr_2$ + CsBr (1:1 ratio)		air	Trace

[a.] Reaction conditions: 2-bromoacetophenone (0.5 mmol, 1.0 equiv.), octanal (1.0 mmol, 2.0 equiv.), Photocatalyst: $CsPbBr_3$ (1.0 mg), CdSe QDs (50 µl 50 µmol/L in hexanes), the other photocatalysts 1.0 mol%; $(ClCH_2CH_2Cl)_2NH$ HCl (0.1 mmol, 20 mol%), 2,6-lutidine (1.0 mmol, 2.0 equiv.) and CH_2Cl_2 (1 mL) under 455 nm blue LED illumination at R.T. [b.] Yield of **1a** determined by ^1H NMR.

Supplementary Table 2. Condition optimization for the synthesis of **1b**.[a]

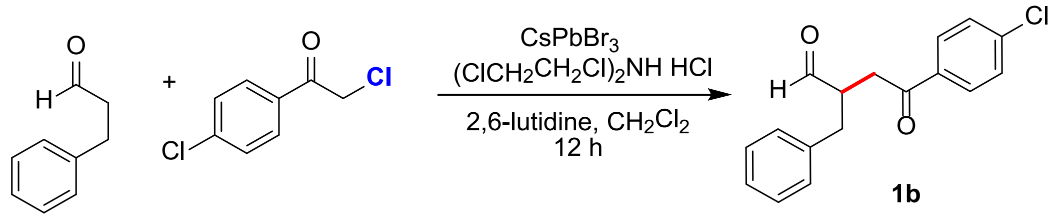

1b

Entry	Variation of conditions	Yield (%) [b]
1	None	52
2	Air-free	50
3	Bulk $CsPbBr_3$ ground powder instead of NCs	<5
4	$PbBr_2$ instead of $CsPbBr_3$ NCs	Trace
5	$PbBr_2$ + CsBr (1:1 ratio) instead of $CsPbBr_3$ NCs	Trace
6	No $CsPbBr_3$	Trace
7	No light	N.R.

[a.] Reaction conditions: 2,4'-dichloroacetophenone (0.5 mmol, 1.0 equiv), octanal (1.0 mmol, 2.0 equiv), $CsPbBr_3$ (1.0 mg), $(ClCH_2CH_2Cl)_2NH$ HCl (0.1 mmol, 20 mol%), 2,6-lutidine (1.0 mmol, 2.0 equiv) and CH_2Cl_2 (1 mL) under 455 nm blue LED illumination at R.T. [b] Yield of **1b**, determined by ^1H NMR.

Supplementary Table 3. Condition optimization for the synthesis of **1c**.[a]

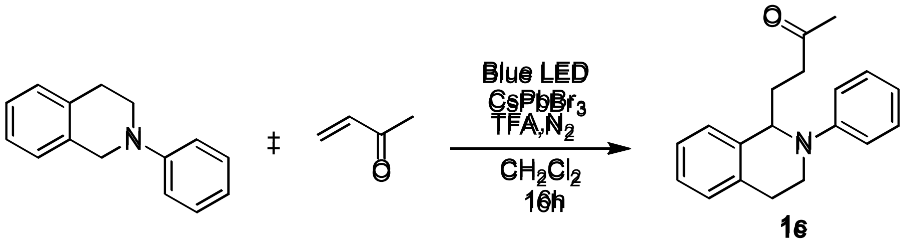

Entry	Variation of conditions	Yield (%) [b]
1	None	90
2	Air	71
3	$ClCH_2CH_2Cl$	83
4	THF	65
5	1,4-dioxane	59
6	Capping ligand oleylamine instead of octylamine	78
7	No TFA	26
8	TFA 50mol %	56
9	TFA 2 equiv.	78
10	TFA 5 equiv.	35
11	$CsPbBr_3$ 0.5 mg	53
12	$CsPbBr_3$ 2.0 mg	89
13	$CsPbBr_3$ ground powder instead of NCs	<5
14	$PbBr_2$ instead of $CsPbBr_3$ NCs	N.R.
15	$PbBr_2$ + CsBr (1:1 ratio) instead of $CsPbBr_3$ NCs	<5
16	$Ru(bpy)_3(PF_6)_2$	60
17	$Ir(ppy)_3$	N.R.
18	525 nm CdSe QDs	N.R.
19	No $CsPbBr_3$	N.R.
20	No light	N.R.

[a.] Reaction conditions: 2-phenyl-1,2,3,4-tetrahydroisoquinoline (0.2 mmol, 1.0 equiv.), 3-buten-2-one (0.4 mmol, 2.0 equiv.), Photocatalyst: $CsPbBr_3$ (1.0 mg), CdSe QDs (50 μl 50 μmol/L in hexanes), the other photocatalysts 1.0 mol%; TFA (0.20 mmol, 1.0 equiv.), and CH_2Cl_2 (2 mL) under 455 nm blue LED illumination at R.T. [b] Yield of **1c** determined by ^1H NMR.

Supplementary Table 4. Condition optimization for the synthesis of **1d**.[a]

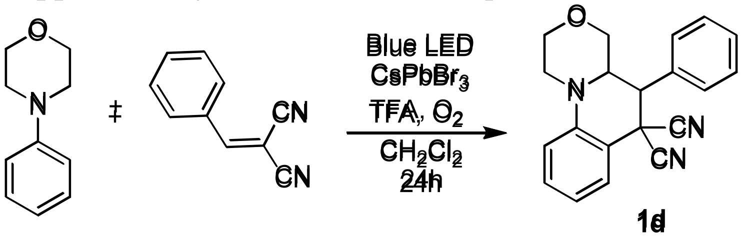

Entry	Variation of conditions	Yield (%) [b]
1	None	79
2	Air-free	<10
3	Air	26
4	$CsPbBr_3$ 0.5 mg	37
5	$CsPbBr_3$ 1.0 mg	73
6	No TFA	15
7	TFA 50 mol%	41
8	TFA 2 equiv.	63
9	TFA 5 equiv.	37
10	Capping ligand oleylamine instead of octylamine	75
11	$CsPbBr_3$ ground powder instead of NCs	<5
12	$PbBr_2$ instead of $CsPbBr_3$ NCs	N.R.
13	$PbBr_2$ + CsBr (1:1 ratio) instead of $CsPbBr_3$ NCs	<5
14	$Ru(bpy)_3(PF_6)_2$	25
15	$Ir(ppy)_3$	N.R.
16	525 nm CdSe QDs	N.R.
17	No $CsPbBr_3$	N.R.
18	No light	N.R.

[a.] Reaction conditions: 2-phenyl-1,2,3,4-tetrahydroisoquinoline (0.2 mmol, 1.0 equiv.), 3-Buten-2-one (0.4mmol, 2.0 equiv.), Photocatalyst: $CsPbBr_3$ (2.0 mg), CdSe QDs (50 µl 50 µmol/L in hexanes), the other photocatalysts (1 mol%), TFA (0.20 mmol, 1.0 equiv.), and CH_2Cl_2 (2 mL) under 455 nm blue LED illumination at R.T. [b] Yield of **1d** determined by ^1H NMR.

Supplementary Table 5. Optimization of reaction condition for the cyclization of benzaldehyde phenylhydrazone with 2-bromoacetophenone.[a]

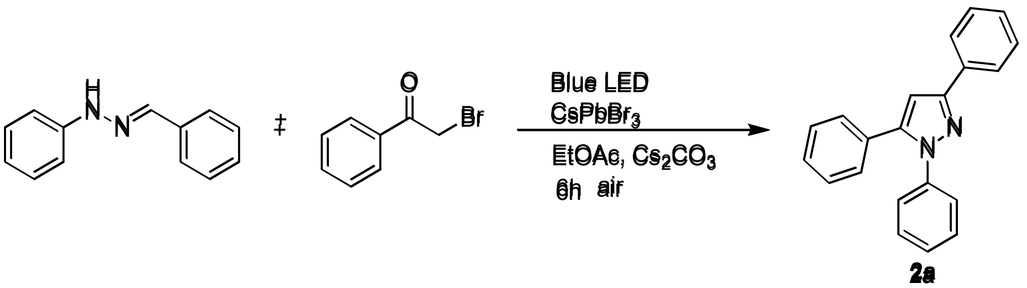

Entry	Variation of conditions	Yield (%)[b]
1	None	86
2	N_2-sparging for 15 min	88
3	CH_2Cl_2 instead of EtOAc	<5
4	THF instead of EtOAc	55
5	$CsPbBr_3$ 0.5 mg	53
6	$CsPbBr_3$ 2.0 mg	88
7	$MAPbBr_3$ instead of $CsPbBr_3$	75
8	$Ir(ppy)_3$	63
9	$Ir(ppy)_3$, N_2-sparging for 15 min	82
10	$Ru(bpy)_3(PF_6)_2$	<5
11	$Ru(bpy)_3(PF_6)_2$, N_2-sparging for 15 min	17
12	CdSe QDs (525 nm) with oleic acid	N.R.
13	CdSe QDs (525 nm) with oleic acid, N_2-sparging for 15 min	N.R.
14	TiO_2 32 nm	N.R.
15	TiO_2 32 nm N_2-sparging for 15 min	N.R.
16	No light	N.R.
17	No base	N.R.
18	No Perovskite	N.R.

[a.] Reaction conditions: 1-benzylidene-2-phenylhydrazine (0.1 mmol, 1.0 equiv.), 2-bromoacetophenone (0.15 mmol, 1.5 equiv.), photocatalyst: $CsPbBr_3$ (1.0 mg); CdSe QDs (50 μl 50 μmol/L in hexanes), the other photocatalysts (1 mol%); base (1.0 equiv.) and solvent (2 mL) under 455 nm blue LED illumination at R.T. without N_2-sparging. [b.] yield determined by ^1H NMR.

Supplementary Table 6. Optimization of reaction condition for the cyclization of ethyl (*Z*)-3-phenyl-3-(phenylamino)acrylate with 2-bromoacetophenone.[a]

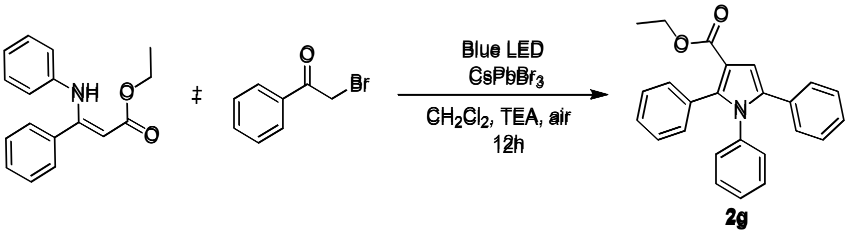

2g

Entry	Variation of conditions	Yield (%)[b]
1	None	93
2	N_2-sparging for 15 min	92
3	1,4-dioxane instead of CH_2Cl_2	<5
4	THF	<5
5	$MAPbBr_3$ instead of $CsPbBr_3$	65
6	$CsPbBr_3$ 0.5 mg	72
7	$CsPbBr_3$ 1.0 mg	89
8	$Ir(ppy)_3$	32
9	$Ir(ppy)_3$, N_2-sparging for 15 min	95
10	$Ru(bpy)_3(PF_6)_2$	12
11	$Ru(bpy)_3(PF_6)_2$, N_2-sparging for 15 min	33
12	CdSe QDs (525 nm) with oleic acid	N.R.
13	CdSe QDs (525 nm) with oleic acid, N_2-sparging for 15 min	8
14	No light	N.R.
15	No base	68
16	No Perovskite	N.R.

[a.] Reaction conditions: ethyl (*Z*)-3-phenyl-3-(phenylamino)acrylate (0.1 mmol, 1.0 equiv.), 2-bromoacetophenone (0.15 mmol, 1.5 equiv.), Photocatalyst: $CsPbBr_3$ (2.0 mg); CdSe QDs (50 μl 50 μmol/L in hexanes), the other photocatalysts (1 mol%); base (35 mol %) and solvent (2 mL) under 455 nm blue LED illumination at R.T. without N_2-sparging. [b.] yield determined by ^1H NMR.

Supplementary Table 7. Optimization of the coupling of benzoic acid with 4-bromotrifluorobenzene.[a]

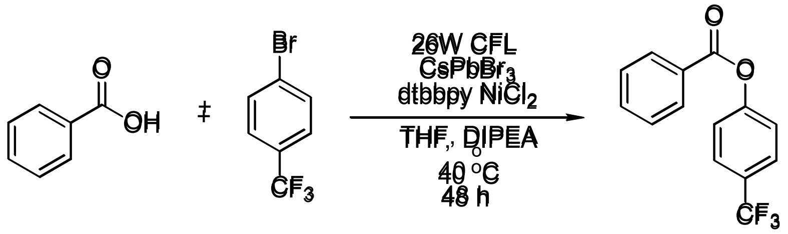

3f

Entry	Variation of conditions	Yield (%)[b]
1	None	72
2	N_2-sparging for 15 min	78
3	$CsPbBr_3$ 1.0 mg	15
4	$CsPbBr_3$ 3.0 mg	56
5	dttbpy $NiBr_2$ instead of dtbbpy $NiCl_2$	<5
6	$CsPbBr_xCl_{3-x}$ (λ_{PL} = 498 nm)	85
7	$CsPbBr_xCl_{3-x}$ (λ_{PL} = 465 nm)	56
8	$CsPbBr_xCl_{3-x}$ (λ_{PL} = 413 nm)	<10
9	$CsPbBr_xI_{3-x}$ (λ_{PL} = 558 nm)	69
10	$CsPbBr_xI_{3-x}$ (λ_{PL} = 600 nm)	15
11	$Ir(ppy)_3$	65
12	$Ir(ppy)_3$, N_2-sparging for 15 min	86
13	$Ru(bpy)_3(PF_6)_2$	N.R.
14	$Ru(bpy)_3(PF_6)_2$, N_2-sparging for 15 min	Trace
15	CdSe QDs (525 nm) with oleic acid	N.R.
16	CdSe QDs (525 nm) with oleic acid, N_2-sparging for 15 min	N.R.
17	No light	N.R.
18	No base	N.R.
19	No Perovskite	N.R.
20	No dtbbpy $NiCl_2$	N.R.

[a.] Performed with photocatalyst: $CsPbBr_3$ (5.0 mg); CdSe QDs (100 μl 50 μmol/L in hexanes), the other photocatalysts (2 mol%); Ni (5 mol%), benzoic acid (0.8 mmol, 2.0 equiv), 4-bromobenzotrifluoride (0.4 mmol, 1.0 equiv), and base (0.8 mmol, 2.0 equiv) under 26 W CFL for 48h without N_2-sparging. [b.] Yields determined by ^1H NMR.

Supplementary Table 8. Reproducibility of the photocatalytic reactions with **P1** in their corresponding optimized conditions.[a]

	Yield (%) [b]					
	**1a**	**1c**	**1d**	**2a**	**2g**	**3f**
**1st**	85	90	79	86	93	72
**2nd**	82	81	80	82	79	67
**3rd**	86	86	71	84	84	71
**Average**	84	85	76	84	85	70

[a.] Conditions: Supplementary Table 1, entry 2 for **1a**; Supplementary Table 3, entry 1 for **1c**; Supplementary Table 4, entry 1 for **1d**; Supplementary Table 5, entry 1 for **2a**; Supplementary Table 6, entry 1 for **2g**; Supplementary Table 7, entry 1 for **3f**.

[b.] Yields determined by ^{1}H NMR.

Supplementary Table 9. Key parameters for $CsPbBr_3$ **P1-P5**.[a]

NCs	First exciton peak (nm)	$E_g(d)$ (eV)	NC size d (nm) [b]	Molecular weight of NC (g mol^{-1})	Surface area-volume ratio (nm^{-1})	Yield (%) [c]	TON for **1a** (based on molecular weight)
**P1**			24	39,960,000	0.25	85	16,983,000
**P2**	515	2.41	14.0	8,015,000	0.43	64	2,565,000
**P3**	506	2.45	8.8	2,375,000	0.68	57	677,000
**P4**	490	2.53	6.0	580,000	1.00	54	157,000
**P5**	456	2.72	3.9	199,000	1.54	8	7900

[a.] The calculation method of key parameters using Supplementary Note 1.

[b.] Size of **P1** was an estimated average size from TEM image.

[c.] Yields from Supplementary Figure 3a.

Supplementary Table 10. Crystallographic data of single crystal for **1d**

	**1d (CCDC 1889861)**
Empirical formula	$C_{20}H_{19}Cl_{0.12}N_3O$
Formula wt	321.81
T, K	273.15
Crystal system	monoclinic
Space group	$P2_1/c$
a, Å	20.198(3)
b, Å	8.9832(15)
c, Å	18.548(3)
α, deg	90
β, deg	109.622(5)
γ, deg	90
V, $Å^3$	3169.9(9)
Z	8
d(cald), Mg m^{-3}	1.349
μ/mm^{-1}	0.105
F(000)	1361.0
Radiation	MoKα (λ = 0.71073)
2θ range, deg	2.14-61.996
Reflections collected	82318
Independent reflections	10088 [R_{int} = 0.0289, R_{sigma} = 0.0174]
Data/restraints/parameters	10088/0/433
goodness-of-fit on F^2	1.018
$R1[I>2\sigma(I)]$	0.0484
$wR2\ [I>2\sigma(I)]$	0.1382

Supplementary Table 11. Selected bond lengths and angles for crystal **1d**.

Atom	Atom	Length/Å	Atom	Atom	Length/Å
C6	C7	1.5305(14)	C19	C0AA	1.5272(15)
C6	C13	1.5619(14)	C2AA	C1AA	1.4058(13)
C6	C7AA	1.5140(14)	C2AA	C1BA	1.5257(14)
C7	C38	1.5309(15)	C2AA	C3BA	1.3938(15)
C7	N3	1.4620(13)	C4	C0AA	1.5308(14)
C13	C22	1.4905(15)	C4	C5AA	1.5207(15)
C13	C24	1.5295(14)	C4	C1BA	1.5569(15)
C13	C34	1.4844(15)	C1AA	C5BA	1.4105(14)
C17	C24	1.3898(15)	C5	C8	1.3890(16)
C17	C11	1.3838(16)	C5	C3BA	1.3813(16)
C22	N25	1.1458(15)	C8	C5BA	1.3841(16)
C24	C14	1.4087(14)	C3AA	C4AA	1.3945(16)
C32	C33	1.3820(16)	C3AA	C5AA	1.4024(16)
C32	C14	1.4071(14)	C4AA	C6AA	1.387(2)
C33	C11	1.3878(17)	C16	C20	1.3921(17)
C34	N39	1.1460(15)	C16	C5AA	1.3973(16)
C38	O0AA	1.4211(13)	C20	C6AA	1.391(2)
C43	C46	1.5056(19)	C8AA	C7AA	1.3962(14)
C43	O0AA	1.4239(15)	C8AA	C9AA	1.3903(15)
C46	N3	1.4648(15)	C0BA	C7AA	1.4006(15)
N3	C14	1.3944(14)	C0BA	C2	1.3907(15)
N40	C3	1.4594(14)	C1BA	C2BA	1.4870(15)
N40	C0AA	1.4669(13)	C1BA	C4BA	1.4818(15)
N40	C1AA	1.3985(13)	C2BA	N2	1.1471(16)
O2	C19	1.4145(14)	N0AA	C4BA	1.1410(15)
O2	C6BA	1.4216(14)	C1	C2	1.3874(16)
C3	C6BA	1.5021(16)	C1	C9AA	1.3877(17)

Supplementary Table 12. Selected bond angles for crystal **1d**.

Atom	Atom	Atom	Angle/°	Atom	Atom	Atom	Angle/°
C7	C6	C13	108.68(8)	C3BA	C2AA	C1BA	118.49(9)
C7AA	C6	C7	113.51(8)	C0AA	C4	C1BA	109.81(8)
C7AA	C6	C13	113.53(8)	C5AA	C4	C0AA	114.26(9)
C6	C7	C38	109.32(8)	C5AA	C4	C1BA	111.77(8)
N3	C7	C6	111.03(8)	N40	C0AA	C19	108.64(8)
N3	C7	C38	107.61(8)	N40	C0AA	C4	110.49(8)
C22	C13	C6	111.57(9)	C19	C0AA	C4	108.30(8)
C22	C13	C24	109.10(8)	N40	C1AA	C2AA	121.55(9)
C24	C13	C6	109.58(8)	N40	C1AA	C5BA	121.62(9)
C34	C13	C6	109.10(8)	C2AA	C1AA	C5BA	116.77(9)
C34	C13	C22	106.53(9)	C3BA	C5	C8	118.51(10)
C34	C13	C24	110.94(9)	C5BA	C8	C5	120.89(10)
C11	C17	C24	121.22(10)	C4AA	C3AA	C5AA	120.83(12)
N25	C22	C13	178.00(12)	C6AA	C4AA	C3AA	119.87(12)
C17	C24	C13	120.02(9)	C20	C16	C5AA	120.57(12)
C17	C24	C14	120.73(9)	C6AA	C20	C16	120.25(12)
C14	C24	C13	119.25(9)	C3AA	C5AA	C4	122.31(10)
C33	C32	C14	121.56(10)	C16	C5AA	C4	119.14(10)
C32	C33	C11	120.70(10)	C16	C5AA	C3AA	118.53(10)
N39	C34	C13	179.56(13)	C4AA	C6AA	C20	119.93(11)
O0AA	C38	C7	112.07(9)	C9AA	C8AA	C7AA	120.62(10)
O0AA	C43	C46	110.97(10)	C2	C0BA	C7AA	120.45(10)
N3	C46	C43	109.70(11)	C8AA	C7AA	C6	118.71(9)
C38	O0AA	C43	110.32(9)	C8AA	C7AA	C0BA	118.69(9)
C7	N3	C46	111.44(9)	C0BA	C7AA	C6	122.58(9)
C14	N3	C7	120.52(9)	C2AA	C1BA	C4	111.92(8)
C14	N3	C46	120.10(9)	C2BA	C1BA	C2AA	110.60(9)
C17	C11	C33	118.75(10)	C2BA	C1BA	C4	106.83(9)
C3	N40	C0AA	111.99(9)	C4BA	C1BA	C2AA	106.92(8)
C1AA	N40	C3	118.38(8)	C4BA	C1BA	C4	113.73(9)
C1AA	N40	C0AA	118.93(8)	C4BA	C1BA	C2BA	106.74(8)
C19	O2	C6BA	109.91(9)	N2	C2BA	C1BA	178.89(14)
N40	C3	C6BA	110.33(9)	C5	C3BA	C2AA	121.31(10)
O2	C19	C0AA	113.80(9)	N0AA	C4BA	C1BA	176.92(13)
C32	C14	C24	117.03(9)	O2	C6BA	C3	111.49(9)
N3	C14	C24	122.08(9)	C8	C5BA	C1AA	121.54(10)
N3	C14	C32	120.72(9)	C2	C1	C9AA	119.81(10)
C1AA	C2AA	C1BA	120.39(9)	C1	C2	C0BA	120.20(10)
C3BA	C2AA	C1AA	120.97(9)	C1	C9AA	C8AA	120.15(10)

Supplementary Note 1 (The calculation of key parameters for $CsPbBr_3$ P1-P5)

Calculation of NC edge length d for P2-P5, while P1 we use 24 nm to estimate.

According to the well-established size-dependent absorbance spectrum of $CsPbBr_3$ NC by equation (1) from Supplementary reference 3.

$$Eg(d) = Eg(\infty) + \frac{1}{(a+bd+rd^2)} \quad (1)$$

Where $E_g(d)$ comes from first exciton of absorption, $E_g(\infty) = 2.25$ eV, $a = -1.26$ eV^{-1}, $b = 0.996$ nm^{-1}eV^{-1}, $r = -0.0324$ nm^{-2}eV^{-1}.

Calculation of numbers of $CsPbBr_3$ units in a NC.

$$\text{NC size/lattice of } CsPbBr_3 = (d/0.583)^3 \quad (2)$$

Calculation of Molecular weight of a NC.

$$\text{Molecular weight} = (d/0.583)^3 \times 579.8 \text{ (g mol}^{-1}\text{)} \quad (3)$$

Calculation of TON based on Molecular weight according to equation (2) and (5).

$$\text{TON} = n_{product}/\{m_{NC}/[(d/0.583)^3 \times 579.8]\} \quad (4)$$

Calculation of Surface area-to-volume ratio:

$$\text{Surface area of one NC} = 6d^2 \text{ (nm}^2\text{)} \quad (5)$$

$$\text{Volume of a NC} = d^3 \text{ (nm}^3\text{)} \quad (6)$$

$$\text{Surface area-to-volume ratio: } 6/d \text{ (nm}^{-1}\text{)} \quad (7)$$

Supplementary Note 2 **(The calculation of TON based on Pb)**

The calculation of TON based on Pb is calculated by the equation (1):

$$\mathrm{TON} = \frac{\text{total mol of product}}{\text{total mol of catalyst}} \qquad (8)$$

Here total mol of catalyst calculated based on Pb.

TON of **1a**:

For a 5.0 mmol scale (10 times scale) reaction **1a**, 1.0 mg **P1** was used, leading to **1a** in 82% yield after 16 hours. After centrifuging the reaction mixture to recover perovskite and to re-apply for a same scale reaction, catalyst is still robust after at least four-times repeats (yield 82%, 80%, 79%, 73%, respectively). TON of $CsPbBr_3$ catalysis is calculated in this way: TON is equal to total mol of product **1a** in 4 repeats of reaction over mol of Pb. TON = (5.0 mmol×(0.82+0.80+ 0.79+ 0.73))/(1.0 mg/ 579.8 g mol^{-1}) ≈ 9,100.

TON of **1c**:

For a large-scale reaction of **1c (**2.0 mmol**)**, 1.0 mg **P1** was used, leading to **1a** in 76% yield after 24 hours. TON = (2.0 mmol×0.71)/(1.0 mg/ 579.8 g mol^{-1}) ≈ 830.

TON of **1d**:

For a large-scale reaction of **1d (**2.0 mmol**)**, 1.0 mg **P1** was used, leading to **1a** in 49% yield after 24 hours. TON = (2.0 mmol×0.49)/(2.0 mg/ 579.8 g mol^{-1}) ≈ 280.

TON of **2a**:

For a 0.2 mmol scale reaction **2a**, 1.0 mg **P1** was used, leading to **2a** in 80% yield after 8 hours. After centrifuging the reaction mixture to recover perovskite and to re-apply for a same scale reaction, catalyst is still robust after at least four-times repeats (yield 83%, 83%, 81%, 79%, respectively). TON = (0.2 mmol×(0.83+0.83+ 0.81+ 0.79))/(1.0 mg/ 579.8 g mol^{-1}) ≈ 380.

TON of **3a**:

For a 0.4 mmol scale reaction **3a**, 5.0 mg **P1** was used, leading to **3a** in 76% yield after 48 hours. TON = (0.4 mmol×0.70)/(5.0 mg/ 579.8 g mol^{-1}) = 33.

Supplementary Note 3 (Emission Quenching Experiments)

Stern-Volmer photoluminescence quenching studies were conducted using 2-phenyl-1,2,3,4-tetrahydro-isoquinoline, 4-phenylmorpholine, (E)-1-benzylidene-2-phenylhydrazine, benzylidenemalononitrile, ethyl (*E*)-3-phenyl-3-(phenylamino)-acrylate and 2,4'-dichloroacetophenone as quenchers.

Rates of quenching (k_q) were determined using Stern-Volmer kinetics by the equation (9):

$$\frac{I_0}{I} = k_q \tau_0 [quencher] \quad (9)$$

Where I_0 is the luminescence intensity without the quencher, I is the intensity with the quencher, and τ_0 is the lifetime of the photocatalyst.

A standard fluorometer setup was used to perform the quenching studies with an excitation wavelength of 455 nm. Samples were prepared by adding solutions of $CsPbBr_3$ NCs (small NCs **P4** instead of **P1**, because more uniform), quenchers, and solvent to obtain a total volume of 2 mL. The concentration of $CsPbBr_3$ NCs **P4** was 0.5 mg mL^{-1}, the same as that of the reaction solution. Samples are irradiated at 455 nm, and the emission intensity at 510 nm is observed, lifetime is ~6 ns.

Supplementary Methods

Radical Trapping Experiments

1) The trapping experiment for the synthesis of 1c

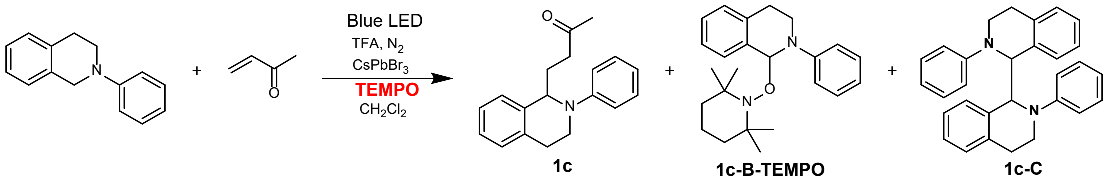

To a 4 mL vial, $CsPbBr_3$ NCs (2.0 mg), 2-phenyl-1,2,3,4-tetrahydroisoquinoline (0.25 mmol, 1.0 equiv.), 3-buten-2-one (0.5 mmol, 2.0 equiv.), TEMPO (0.5 mmol, 2.0 equiv.), trifluoroacetic acid (0.05 mmol, 20 mol%), and 2 mL DCM, the mixture was bubbled with N_2 for 10 min and then stirred. After irradiation with Blue LED for 8h, trace amount of **1c** and **1c-C** was isolated, while **1d-B-TEMPO** was detected by LC-MS. The crude self-coupling product **1c-C** was purified by column chromatography to afford a mixture of diastereoisomers with 1.28:1 dr. ^{1}H NMR (500 MHz, $CDCl_3$) δ 7.39-6.86 (m, 16H, Ar**H** (major+minor)), 6.79 (t, 2H, Ar**H** (minor)), 6.75 (t, 2H, Ar**H** (major)), 5.37 (s, 2H, C**H**C**H**, major), 5.34 (s, 2H, C**H**C**H**, minor), 3.56-3.52 (m, 2H, NC**H**$_2$CH$_2$, minor), 3.42-3.25 (m, 4H, NC**H**$_2$CH$_2$, major+minor), 2.87-2.82 (m, 2H, PhC**H**$_2$CH$_2$, major), 2.69-2.59 (m, 2H, PhC**H**$_2$CH$_2$, major+minor), 2.08-2.02 (m, 2H, PhC**H**$_2$CH$_2$, minor). ESI-MS m/z calcd for $C_{30}H_{29}N_2^+$ ($[M+H]^+$) 417.2331, found: 417.2335.

2) The trapping experiment for the synthesis of 1d

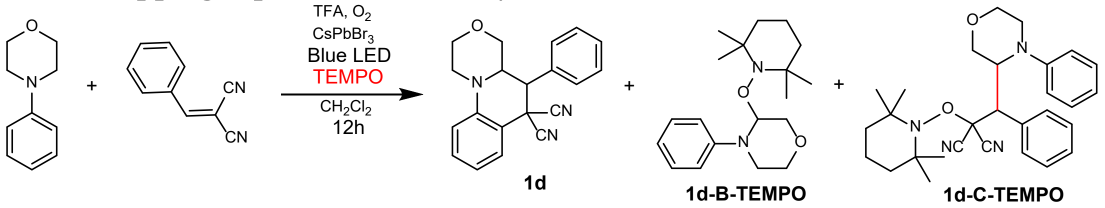

To an 8 mL vial, $CsPbBr_3$ NCs (2.0 mg), 4-phenylmorpholine (0.25 mmol,1.0 equiv.), 2-benzylidenemalononitrile (0.5 mmol, 2.0 equiv.), trifluoroacetic acid (0.05 mmol, 20 mol%), and 1 mL DCM were added, the mixture was bubbled with O_2 for 10 min and then stirred. After irradiation with Blue LED for 8h, trace amount of **1d-B-TEMPO** and **1d-C-TEMPO** was detected by LC-MS.

3) The trapping experiment for the synthesis of 2a

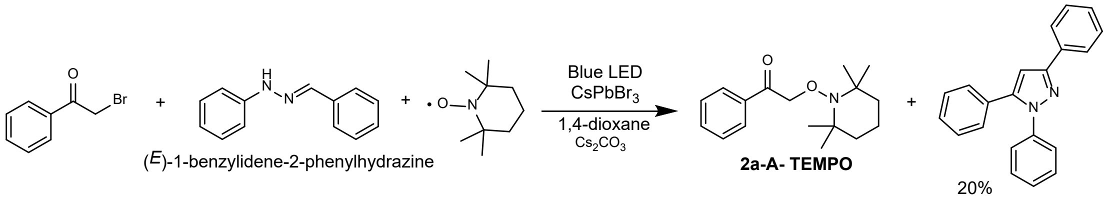

In a 4 mL vial equipped with (*E*)-1-benzylidene-2-phenylhydrazine (0.1 mmol), 2-bromoacetophenone (0.15 mmol), TEMPO (0.3 mmol), TEA (5 μL), $CsPbBr_3$ Perovskite NCs **P1** (2.0 mg), and 1 mL DCM were added and then stirred under the irradiation with blue LED lamp for 24 h. Afford **2a** with 20% yield, along the trapping product **2a-A-TEMPO** was isolated and confirmed by ^{1}H NMR.

4) The trapping experiment of 2a-C

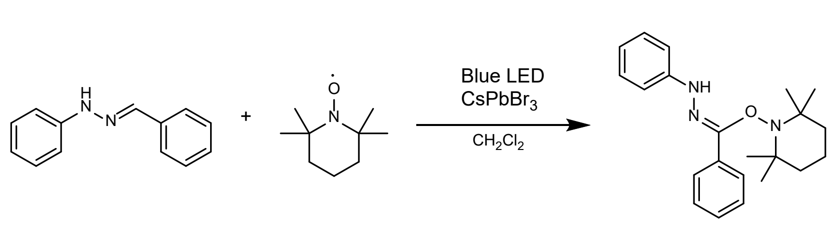

2a-C-TEMPO

In a 4 mL vial equipped with (E)-1-benzylidene-2-phenylhydrazine (0.1 mmol), TEMPO (0.2 mmol), $CsPbBr_3$ Perovskite NCs **P1** (2.0 mg), and 1 mL CH_2Cl_2 were added and then stirred under the irradiation with blue LED lamp for 24 h. The trapping products **2a-C-TEMPO** was detected by LC-MS.

5) The trapping experiment for the synthesis of 2g

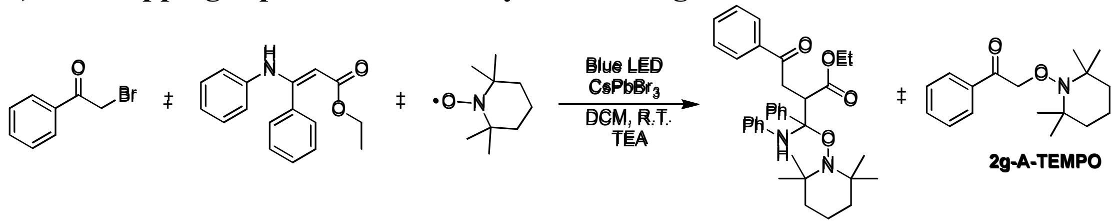

2g-A-TEMPO

2g-C-TEMPO

In a 4 mL vial equipped with ethyl (*E*)-3-phenyl-3-(phenylamino)acrylate (0.1 mmol), 2-bromoacetophenone (0.15 mmol), TEMPO (0.3 mmol), TEA (5 μL), $CsPbBr_3$ Perovskite NCs **P1** (2.0 mg), and 1 mL DCM were added and then stirred under the irradiation with blue LED lamp for 24 h. Two trapping products **2g-A-TEMPO** and **2g-C-TEMPO** were detected by LC-MS.

Synthesis of key precursors

Substituted benzylidene phenylhydrazones and ethyl (*Z*)-3-phenyl-3-(phenylamino)acrylate were prepared according to literature procedures.[4]

Photocatalytic organic synthesis procedure

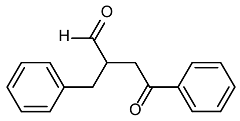

2-Benzyl-4-oxo-4-phenylbutanal (1a)

The crude product was purified by column chromatography (silica gel, Hexane/EtOAc = 10:1) to afford white solid, yield: 85 %). ^1H NMR (500 MHz, $CDCl_3$) δ 9.93 (s, 1H), 7.93 (d, J = 7.5 Hz, 2H), 7.59 (t, J = 7.5 Hz, 2H), 7.47 (t, J = 7.5 Hz, 2H), 7.34-7.22 (m, 5H), 3.47-3.41 (m, 2H), 3.20 (dd, J_1 = 14.0 Hz, J_2 = 6.0 Hz, 1H), 3.06-3.02 (m, 1H), 2.85 (dd, J_1 = 14.0 Hz, J_2 = 6.0 Hz, 1H). The characterization data are consistent with our previous report.[5]

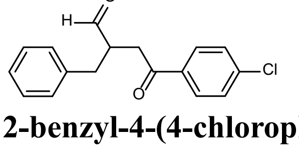

2-benzyl-4-(4-chlorophenyl)-4-oxobutanal (1b)

The crude product was purified by column chromatography (silica gel, Hexane/EtOAc = 10:1) to afford white solid, yield: 80 % for 2-bromo-4'-chloroacetophenone as substrate; 52 % for 2,4'-dichloro-acetophenone as substrate). ^1H NMR (400 MHz, $CDCl_3$) δ 9.89 (s, 1H), 7.83 (d, J = 8.4 Hz, 2H), 7.41 (d, J = 8.4 Hz,

2H), 7.31-7.28 (m, 2H), 7.26-7.18 (m, 3H), 3.42-3.34 (m, 2H), 3.17 (dd, J_1 = 14.0 Hz, J_2 = 6.0 Hz, 1H), 2.98-2.91 (m, 1H), 2.81 (dd, J_1 = 14.0 Hz, J_2 = 8.4 Hz, 1H). ^{13}C NMR (100 MHz, $CDCl_3$) δ 202.80, 196.67, 139.79, 137.91, 134.72, 129.47, 128. 99, 128.92, 128.79, 16.81, 48.38, 37.05, 34.65. ESI-MS m/z calcd for $C_{17}H_{15}ClO_2Na^+$ ($[M+Na]^+$) 309.0658, found: 309.0672.

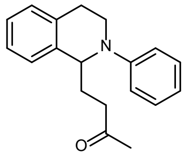

Synthesis of 4-(2-Phenyl-1,2,3,4-tetrahydroisoquinolin-1-yl)butan-2-one (1c)

To a 4 mL vial, $CsPbBr_3$ NCs **P1** (2.0 mg), 2-phenyl-1,2,3,4-tetrahydroisoquinoline (0.2 mmol, 1.0 equiv.), 3-buten-2-one (0.4 mmol, 2.0 equiv.), trifluoroacetic acid (0.20 mmol, 1.0 equiv.), and 2 mL DCM, , the mixture was bubbled with N_2 for 10 min and then stirred under the irradiation with a 12 W 455 nm Blue LED lamp, distance ~ 8 cm. After 16h, the mixture was poured into water, and extracted with Et_2O (3×10 mL). The combined organic layers were washed with water, dried over Na_2SO_4 and concentrated *in vacuo*. The crude product was purified by column chromatography (silica gel, Hexane/EtOAc = 10:1) to afford colorless oil 99 mg, yield: 90 %). ^{1}H NMR (500 MHz, $CDCl_3$) δ 7.28-7.24 (m, 2H), 7.20-7.11 (m, 3H), 6.91 (d, J = 8 Hz, 2H), 6.77 (t, J = 7.5 Hz, 1H), 4.76 (dd, J_1 = 9.5 Hz, J_2 = 6.0 Hz, 1H), 3.68-3.55 (m, 2H), 3.06-3.00 (m, 1H), 2.78 (dt, J_1 = 16.5 Hz, J_2 = 4.5 Hz, 1H), 2.60 (t, J = 7.0 Hz, 2H), 2.30-2.22 (m, 1H), 2.12 (s, 3H), 2.11-2.05 (m, 1H). The characterization data are consistent with those reported in the literature.[6]

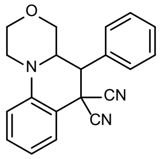

Synthesis of 5-Phenyl-1,2,4a,5-tetrahydro-[1,4]oxazino[4,3-a]quinoline-6,6(4H)-dicarbonitrile (1d)

To an 8 mL vial, $CsPbBr_3$ NCs **P1** (2.0 mg), 4-phenylmorpholine (0.20 mmol,1.0 equiv.), 2-benzylidenemalononitrile (0.5 mmol, 2.0 equiv.), trifluoroacetic acid (0.20 mmol, 1.0 equiv., and 2 mL DCM were added, the mixture was bubbled with O_2 for 10 min and then stirred under the irradiation with a 12 W 455 nm Blue LED lamp, distance ~ 8 cm. After 24h, the mixture was poured into water, and extracted with Et_2O (3 × 10 mL). The combined organic layers were washed with water, dried over Na_2SO_4 and concentrated *in vacuo*. The crude product was purified by column chromatography (silica gel, Hexane/EtOAc = 10:1) to afford white solid, yield: 73 %). ^{1}H NMR (500 MHz, $CDCl_3$) δ 7.56-7.45 (m, 6H), 7.40-7.36 (m, 1H), 6.97-6.84 (m, 2H), 4.03 (dd, J_1 = 14.0 Hz, J_2 = 4.5 Hz, 1H), 3.87 (td, J_1 = 13.0 Hz, J_2 = 4.0 Hz, 1H), 3.78 (d, J = 16.0 Hz, 1H), 3.73-3.64 (m, 2H), 3.28 (d, J = 13.5 Hz, 1H), 3.21-3.13 (m, 2H). Crystal Data for $C_{20}H_{19}Cl_{0.12}N_3O$ (M =321.81 g mol^{-1}): monoclinic, space group P2$_1$/c (no. 14), a = 20.198(3) Å, b = 8.9832(15) Å, c = 18.548(3) Å, β = 109.622(5), V = 3169.9(9) Å^3, Z = 8, T = 273.15 K, μ(MoKα) = 0.105 mm^{-1}, $Dcalc$ = 1.349 g cm^{-3}, 82318 reflections measured (2.14° ⩽ 2θ ⩽ 61.996°), 10088 unique (R_{int} = 0.0289, R_{sigma} = 0.0174) which were used in all calculations. The final R_1 was 0.0484 (I > 2σ(I)) and wR_2 was 0.1382 (all data).

General procedure for the synthesis of pyrazoles (2a-2f)

To a 4 mL vial, $CsPbBr_3$ Perovskite **P1** (2.0 mg), benzylidene phenylhydrazone (0.1 mmol, 1 equiv.), the corresponding bromide (0.15 mmol, 1.5 equiv.), Cs_2CO_3 (0.15 mmol, 1.5 equiv.), and 2 mL 1,4-dioxane were added and then stirred under the irradiation with a 12 W 455 nm Blue LED lamp, distance ~ 8 cm. After the reaction was complete (monitored via TLC, ~ 6-12 h), the mixture was poured into water, and extracted with CH_2Cl_2 (3 × 10 mL). The combined organic layers were washed with water, dried over Na_2SO_4 and concentrated *in vacuo*. Purification of the crude product by column chromatography (silica gel) to afford the desired product.

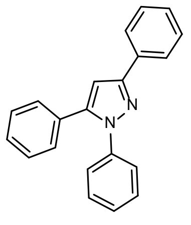

1,3,5-Triphenyl-1H-pyrazole (2a)

The crude product was purified by column chromatography (silica gel, Hexane/EtOAc = 20:1) to afford white solid: 25.4 mg, yield: 83%. ^{1}H NMR (500 MHz, $CDCl_3$) δ 7.95 (d, J = 8.0 Hz, 2H), 7.45 (t, J = 7.5 Hz, 2H), 7.41-7.29 (m, 11H), 6.86 (s, 1H). The characterization data are consistent with those reported in the literature.[7]

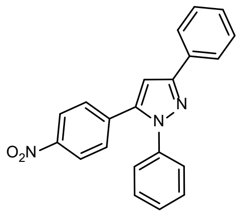

5-(4-nitrophenyl)-1,3-diphenyl-1H-pyrazole (2b)

The crude product was purified by column chromatography (silica gel, Hexane/EtOAc = 20:1) to afford white solid: 27.4 mg, yield: 84%. ^{1}H NMR (500 MHz, $CDCl_3$) δ 8.20 (d, J = 7.0 Hz, 2H), 7.94 (d, J = 8.0 Hz, 2H), 7.49-7.37 (m, 10H), 6.97 (s, 1H). The characterization data are consistent with those reported in the literature.[7]

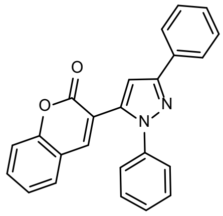

3-(1,3-Diphenyl-1H-pyrazol-5-yl)-2H-chromen-2-one (2c)

The crude product was purified by column chromatography (silica gel, Hexane/EtOAc = 20:1) to afford yellow solid: 33.0 mg, yield: 89%. ^{1}H NMR (500 MHz, $CDCl_3$) δ 7.93 (d, J = 8.0 Hz, 2H), 7.58-7.51 (m, 4H), 7.46-7.42 (m, 4H), 7.39-7.34 (m, 4H), 7.30-7.26 (m, 1H), 7.14 (s, 1H). ^{13}C NMR (125 MHz, $CDCl_3$) δ 161.50, 156.30, 154.80, 145.15, 145.08, 142.93, 139.77, 135.37, 134.97, 132.02, 131.31, 130.80, 130.66, 128.57, 128.51, 121.38, 121.38, 121.24, 110.37, 110.32. ESI-MS m/z calcd for $C_{24}H_{17}N_2O_2^+$ ($[M+H]^+$) 365.1290, found: 365.1276.

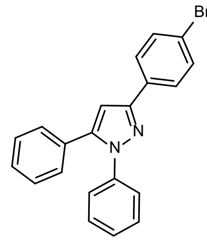

3-(4-Bromophenyl)-1,5-diphenyl-1H-pyrazole (2d)

The crude product was purified by column chromatography (silica gel, Hexane/EtOAc = 20:1) to afford white solid: 31.2 mg, yield: 83%. ^{1}H NMR (500 MHz, $CDCl_3$) δ 7.82 (d, J = 8.5 Hz, 2H), 7.58 (d, J = 8.5 Hz, 2H), 7.39-7.29 (m, 10H), 6.82 (s, 1H). The characterization data are consistent with those reported in the literature.[7]

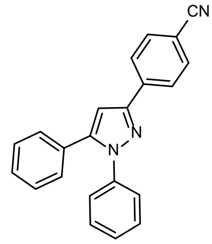

4-(1,5-Diphenyl-1H-pyrazol-3-yl)benzonitrile (2e)

The crude product was purified by column chromatography (silica gel, Hexane/EtOAc = 20:1) to afford yellow solid: 28.8 mg, yield: 90%. ^{1}H NMR (500 MHz, $CDCl_3$) δ 8.05 (d, J = 8.0 Hz, 2H), 7.70 (d, J = 8.0 Hz, 2H), 7.40-7.36 (m, 8H), 7.36-7.29 (m, 2H), 6.89 (s, 1H). The characterization data are consistent with those reported in the literature.[7]

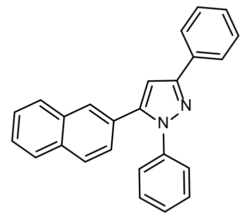

5-(Naphthalen-2-yl)-1,3-diphenyl-1H-pyrazole (2f)

The crude product was purified by column chromatography (silica gel, Hexane/EtOAc = 20:1) to afford white solid: 28.0 mg, yield: 82%. ^{1}H NMR (500 MHz, $CDCl_3$) δ 7.99 (d, J = 8.0 Hz, 2H), 7.89 (s, 1H), 7.86-7.77 (m, 3H), 7.54-7.44 (m, 6H), 7.38-7.31 (m, 5H), 6.97 (s, 1H). The characterization data are consistent with those reported in the literature.[7]

General procedure for the synthesis of pyrroles (2g-2i)

To a 4 mL vial, $CsPbBr_3$ Perovskite **P1** (2.0 mg), Ethyl (Z)-3-phenyl-3-(phenylamino)acrylate (0.1 mmol, 1.0 equiv), the corresponding bromide (0.15 mmol, 1.5 equiv), TEA (0.035 mmol, 35mol %), and 2 mL CH_2Cl_2 were added and then stirred under the irradiation with a 12 W 455 nm Blue LED lamp, distance ~ 8 cm. After the reaction was complete (monitored via TLC), the mixture was poured into water, and extracted with CH_2Cl_2 (3 × 10 mL). The combined organic layers were washed with water, dried over Na_2SO_4 and concentrated *in vacuo*. Purification of the crude product by column chromatography (silica gel) to afford the desired product.

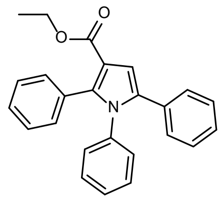

Ethyl 1,2,5-triphenyl-1H-pyrrole-3-carboxylate (2g)

White solid: 35.0 mg, yield: 95%. ^{1}H NMR (500 MHz, $CDCl_3$) δ 7.24-7.17 (m, 11H), 7.12-7.10 (m, 2H), 6.95 (s, 1H), 6.93-6.90 (m, 2H), 4.17 (q, J = 7.0 Hz, 2H), 1.17 (t, J = 7.0 Hz, 3H). The characterization data are consistent with those reported in the literature.[8]

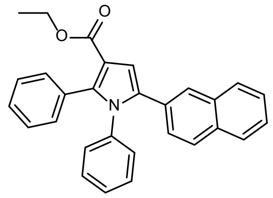

Ethyl 5-(naphthalen-2-yl)-1,2-diphenyl-1H-pyrrole-3-carboxylate (2h)

The crude product was purified by column chromatography (silica gel, Hexane/EtOAc = 10:1) to afford white solid: 32.7 mg, yield: 78%. ^{1}H NMR (500 MHz, $CDCl_3$) δ 7.76 (m, 1H), 7.75-7.61 (m, 3H), 7.45-7.43 (m, 2H), 7.25-7.15 (m, 8H), 7.09 (s, 1H), 7.00-6.98 (m, 2H), 4.21 (q, J = 7.0 Hz, 2H), 1.22 (t, J = 7.0 Hz, 3H). The characterization data are consistent with those reported in the literature.[8]

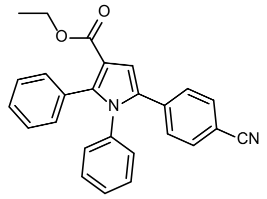

Ethyl 5-(4-cyanophenyl)-1,2-diphenyl-1H-pyrrole-3-carboxylate (2i)

The crude product was purified by column chromatography (silica gel, Hexane/EtOAc = 10:1) to afford white solid: 24.7 mg, yield: 63%. ^{1}H NMR (500 MHz, $CDCl_3$) δ 7.47 (d, J = 8.0 Hz, 1H), 7.28-7.22 (m, 6H), 7.20-7.16 (m, 4H), 7.10 (s, 1H), 6.94 (d, J = 8.0 Hz, 2H), 4.20 (q, J = 7.0 Hz, 2H), 1.19 (t, J = 7.0 Hz, 3H). The characterization data are consistent with those reported in the literature.[8]

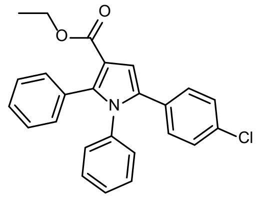

Ethyl 5-(4-chlorophenyl)-1,2-diphenyl-1*H*-pyrrole-3-carboxylate (2j)

To a 4 mL vial, $CsPbBr_3$ **P1** (3.0 mg), ethyl (Z)-3-phenyl-3-(phenylamino)acrylate (0.1 mmol, 1.0 equiv.), 2,4'-dichloroacetophenone (0.15 mmol, 1.5 equiv.), TEA (0.035 mmol, 35mol %), and 2 mL 1,4-dioxane were added and then stirred under the irradiation with a 12 W 455 nm blue LED lamp, distance ~ 8 cm. After irradiation for 24 h, the mixture was poured into water, and extracted with CH_2Cl_2 (3 × 10 mL). The combined organic layers were washed with water, dried over Na_2SO_4 and concentrated *in vacuo*. Purification of the crude product by column chromatography (silica gel,

Hexane/EtOAc = 10:1) to afford white solid: 26.7 mg, yield: 67%. ^{1}H NMR (400 MHz, $CDCl_3$) δ 7.23-7.12 (m, 10H), 7.01-6.98 (m, 2H), 6.94 (s, 1H), 6.92-6.89 (m, 2H), 4.18 (q, J = 7.2 Hz, 2H), 1.71 (t, J = 7.2 Hz, 3H). ^{13}C NMR (100 MHz, $CDCl_3$) δ 164.58, 140.12, 137.64, 133.42, 132.76, 131.43, 131.23, 130.67, 129.69, 128.75, 128.74, 128.68, 128.27, 128.24, 127.82, 127.79, 127.25, 114.41, 111.11, 59.67, 14.18. ESI-MS m/z calcd for $C_{25}H_{21}ClNO_2^+$ ($[M+1]^+$) 402.1261, found: 402.1266.

General procedure of the couplings of carboxylic acid and aryl bromides (3a-3f)

To a 4 mL vial, 5.0 mg $CsPbBr_3$ **P1** carboxylic acid (0.8 mmol, 2.0 equiv), aryl bromide (0.4 mmol, 1.0 equiv), dtbbpy·Ni(II)Cl_2 (8.0 mg, 0.02 mmol, 0.05 equiv), and DIPEA (139 μL, 0.8 mmol, 2.0 equiv) were added into 2 mL THF. The mixture was stirred at 40 °C under the irradiation with two 26 W compact fluorescent lights, distance ~ 5 cm. After 48 h, the mixture was poured into water, and extracted with EtOAc (3 × 10 mL). The combined organic layers were washed with water, dried over Na_2SO_4 and concentrated *in vacuo*. Purification of the crude product by column chromatography (silica gel) to afford the desired product.

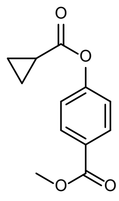

Methyl 4-((cyclopropanecarbonyl)oxy)benzoate (3a)

The crude product was purified by column chromatography (silica gel, Hexane/EtOAc = 20:1) to afford 66 mg white solid, yield: 72 %. ^{1}H NMR (500 MHz, $CDCl_3$) δ 8.08 (d, J = 8.5 Hz, 2H), 7.20 (d, J = 9.0 Hz, 2H), 3.93 (s, 3H), 1.89-1.85 (m, 1H), 1.22-1.19 (m, 2H), 1.09-1.05 (m, 2H). The characterization data are consistent with those reported in the literature.[9]

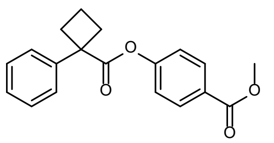

Methyl 4-((1-phenylcyclobutane-1-carbonyl)oxy)benzoate (3b)

The crude product was purified by column chromatography (silica gel, Hexane/EtOAc = 10:1) to afford white solid, yield: 74 %. ^{1}H NMR (500 MHz, $CDCl_3$) δ 8.04 (d, J = 8.5 Hz, 2H), 7.45-7.40 (m, 4H), 7.32 (t, J = 6.5 Hz, 2H), 7.05 (d, J = 9.0 Hz, 2H), 3.92 (s, 3H), 3.05-2.99 (m, 2H), 2.71-2.65 (m, 2H), 2.21-2.14 (m, 1H), 2.04-1,97 (m, 1H). ^{13}C NMR (125 MHz, $CDCl_3$) δ 173.88, 166.33, 154.77, 142.65, 131.05, 128.54, 127.56, 127.03, 126.35, 121.35, 52.67, 52.18, 32.26, 16.68. ESI-MS m/z calcd for $C_{19}H_{18}O_4Na^+$ ($[M+Na]^+$) 333.1103, found: 333.1135.

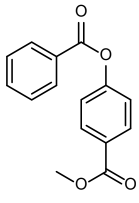

Methyl 4-(benzoyloxy)benzoate (3c)

The crude product was purified by column chromatography (silica gel, Hexane/EtOAc = 10:1) to afford white solid, yield: 76 %. ^{1}H NMR (500 MHz, $CDCl_3$)

δ 8.23 (d, J = 8.0 Hz, 2H), 8.15 (d, J = 8.5 Hz, 2H), 7.68 (t, J = 7.5 Hz, 1H), 7.55 (t, J = 7.5 Hz, 2H), 7.33 (d, J = 8.5 Hz, 2H), 3.96 (s, 3H). The characterization data are consistent with those reported in the literature.[1]

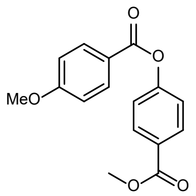

4-(Methoxycarbonyl)phenyl 4-methoxybenzoate (3d)

The crude product was purified by column chromatography (silica gel, Hexane/EtOAc = 10:1) to afford white solid, yield: 69 %. ^{1}H NMR (500 MHz, $CDCl_3$) δ 8.17 (d, J = 9.0 Hz, 2H), 8.14 (d, J = 9.0 Hz, 2H), 7.32 (d, J = 8.5 Hz, 2H), 7.02 (d, J = 8.5 Hz, 2H), 3.95 (s, 3H), 3.93 3.95 (s, 3H). The characterization data are consistent with those reported in the literature.[1]

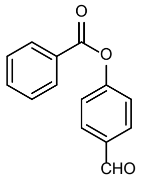

4-Formylphenyl benzoate (3e)

The crude product was purified by column chromatography (silica gel, Hexane/EtOAc = 20:1) to afford white solid, yield: 75 %. ^{1}H NMR (500 MHz, $CDCl_3$) δ 10.02 (s, 1H), 8.21 (d, J = 7.0 Hz, 2H), 7.97 (d, J = 8.5 Hz, 2H), 7.66 (t, J = 7.5 Hz, 1H), 7.53 (t, J = 8.0 Hz, 2H), 7.41 (d, J = 8.5 Hz, 2H). The characterization data are consistent with those reported in the literature.[1]

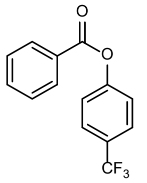

4-(Trifluoromethyl)phenyl benzoate (3f)

The crude product was purified by column chromatography (silica gel, Hexane/EtOAc = 20:1) to afford 89 mg white solid, yield: 84 %. ^{1}H NMR (500 MHz, $CDCl_3$) δ 8.24 (d, J = 7.0 Hz, 2H), 7.73 (d, J = 9.0 Hz, 2H), 7.70 (t, J = 7.5 Hz, 1H), 7.56 (t, J = 7.5 Hz, 2H), 7.39 (d, J = 8.5 Hz, 2H). The characterization data are consistent with those reported in the literature.[1]

Supplementary References

1. Welin, E. R. *et al.* Photosensitized, energy transfer-mediated organometallic catalysis through electronically excited nickel(II). *Science* **355**, 380-385 (2017).

2. Caputo, J. A., Frenette, L. C., Zhao, N., Sowers, K. L., Krauss, T. D. & Weix, D. General and Efficient C–C Bond Forming Photoredox Catalysis with Semiconductor Quantum Dots. *J. Am. Chem. Soc.* **139**, 4250-4253 (2017).

3. Maes, J. *et al.* Light Absorption Coefficient of $CsPbBr_3$ Perovskite Nanocrystals. *J. Phys. Chem. Lett.* **9**, 3093-3097 (2018).

4. Wurtz, S. *et al.* Palladium-catalyzed oxidative cyclization of *N*-aryl enamines: from anilines to indoles. *Angew. Chem., Int. Ed.* **47**, 7230-7233 (2008).

5. Zhu, X., Lin, Y., Sun, Y., Beard, M. C. & Yan, Y. Lead-Halide Perovskites for Photocatalytic α-Alkylation of Aldehydes. *J. Am. Chem. Soc.* **141**, 733-738 (2019).

6. Espelt, L. R., Wiensch, E. M., Yoon, T. P. Brønsted Acid Cocatalysts in Photocatalytic Radical Addition of α-Amino C-H Bonds across Michael Acceptors. *J. Org. Chem.* **78**, 4107-4114 (2013).

7. Fan, X.-W. Radical Addition of Hydrazones by *α*-Bromo Ketones to Prepare 1,3,5-Trisubstituted Pyrazoles via Visible Light Catalysis. *J. Org. Chem.* **81**, 7127-7133 (2016).

8. Lei, T. *et al.* Radical Visible Light Initiated Hantzsch Synthesis of 2,5-Diaryl-Substituted Pyrroles at Ambient Conditions. *Org. Lett.* **14**, 2479-2482 (2016).
