# Supplementary material for: Lead halide perovskites for photocatalytic organic synthesis
Source: Nat Commun. 2019 Jun 28;10:2843. doi: 10.1038/s41467-019-10634-x (PMC6599021; doi:10.1038/s41467-019-10634-x)
Supplement: Supplementary file 3 — Description of Additional Supplementary Information [file 41467_2019_10634_MOESM3_ESM.pdf]

Description of Additional Supplementary Files

File Name: Supplementary Movie 1
Description: $CsPbBr_3$ NCs **P1** PL intensity increasing visualized after addition of 5 μl trifluoroacetic acid.
